# Controlled Infection Immunization Using Delayed Death Drug Treatment Elicits Protective Immune Responses to Blood-Stage Malaria Parasites

**DOI:** 10.1128/IAI.00587-18

**Published:** 2018-12-19

**Authors:** Leanne M. Low, Aloysious Ssemaganda, Xue Q. Liu, Mei-Fong Ho, Victoria Ozberk, James Fink, Lana Sundac, Kylie Alcorn, Amy Morrison, Kevin O’Callaghan, John Gerrard, Danielle I. Stanisic, Michael F. Good

**Affiliations:** a Institute for Glycomics, Griffith University, Gold Coast Campus, Queensland, Australia; b Gold Coast University Hospital, Southport, Queensland, Australia; University of South Florida

**Keywords:** malaria

## Abstract

Naturally acquired immunity to malaria is robust and protective against all strains of the same species of *Plasmodium*. This develops as a result of repeated natural infection, taking several years to develop. Evidence suggests that apoptosis of immune lymphocytes due to uncontrolled parasite growth contributes to the slow acquisition of immunity. To hasten and augment the development of natural immunity, we studied controlled infection immunization (CII) using low-dose exposure to different parasite species (Plasmodium chabaudi, P. yoelii, or P. falciparum) in two rodent systems (BALB/c and C57BL/6 mice) and in human volunteers, with drug therapy commencing at the time of initiation of infection. CIIs with infected erythrocytes and in conjunction with doxycycline or azithromycin, which are delayed death drugs targeting the parasite’s apicoplast, allowed extended exposure to parasites at low levels. In turn, this induced strong protection against homologous challenge in all immunized mice. We show that P. chabaudi*/*P. yoelii infection initiated at the commencement of doxycycline therapy leads to cellular or antibody-mediated protective immune responses in mice, with a broad Th1 cytokine response providing the best correlate of protection against homologous and heterologous species of *Plasmodium*. P. falciparum CII with doxycycline was additionally tested in a pilot clinical study (*n* = 4) and was found to be well tolerated and immunogenic, with immunological studies primarily detecting increased cell-associated immune responses. Furthermore, we report that a single dose of the longer-acting drug, azithromycin, given to mice (*n* = 5) as a single subcutaneous treatment at the initiation of infection controlled P. yoelii infection and protected all mice against subsequent challenge.

## Abstract (precis)

Naturally acquired immunity to malaria is robust and protective against all strains of the same species of *Plasmodium*. This develops as a result of repeated natural infection, taking several years to develop.

## INTRODUCTION

Malaria is a disease of worldwide importance, with an estimated 445,000 deaths being caused by *Plasmodium* spp. in 2016 (1). While naturally acquired immunity (NAI; a state of partial nonsterilizing immunity) can develop in individuals living in areas where malaria is endemic, this takes several years to develop (2–4). This delay is believed to be due to not only the malaria parasite’s ability to alter its surface antigens (5, 6) and age-related differences in immune responses (2, 7) but also the exhaustion of T and B lymphocytes during chronic infection (8, 9). Chronic or high parasite density is a critical factor leading to apoptosis of parasite-specific effector T cells (10–12). T cell exhaustion and apoptosis are critical, as T cells, if functional, can control parasite growth, particularly as they often recognize epitopes that are highly conserved between parasite strains (10, 11, 13). It is consistent with these observations that an individual requires multiple infections to build and sustain a repertoire of protective immune responses (4, 14).

In more recent years, controlled infection immunization (CII) was shown to mimic the acquisition of natural immunity without prolonged infection. This approach, whereby infection is initiated but then controlled by drugs, has been studied extensively with sporozoites (in rodents, nonhuman primates, and humans), demonstrating strong protection against homologous challenge (15–24) and limited protection against heterologous challenge (25). Cross-stage protection was also observed in rodent models (17, 20); however, little to no blood-stage protection was noted in humans (23). Thus, parasites that are able to evade liver-stage immunity can establish an uncontrolled blood-stage infection.

CII was studied using blood-stage malaria parasites, although less extensively (10, 26, 27). In these studies, treatment with atovaquone-proguanil or chloroquine was administered after a certain period of time (e.g., 48 h to allow 2 cycles of replication in rodents [10] and 8 days to allow 4 cycles in humans [26]), and protective immune responses were demonstrated against cross-stage (sporozoites) (27) and homologous (10, 26) and heterologous (10) blood-stage parasites. The protection against clinical malaria observed in the human study had to be qualified, as it was subsequently shown that the drug used to treat the volunteers had a longer than expected half-life and residual drug may have contributed to the observed protection (28). Nevertheless, strong parasite-specific cellular immune responses were observed, including the production of gamma interferon (IFN-γ) and nitric oxide synthase (26). An association of protection with the production of IFN-γ, tumor necrosis factor (TNF), and interleukin-2 (IL-2) by T cells following parasite stimulation has been noted in various studies (10, 27, 29–37). IFN-γ and TNF have also been associated with NAI in humans (38–41); additionally, protective antibody responses to blood-stage parasites are well recognized in NAI (42, 43).

In considering the feasibility of a CII vaccination approach using blood-stage parasites, two safety issues arise: first, drug treatment must be initiated at the time of infection and not after a delayed period, and second, a single dose of drug, not multiple doses given over an extended period, is required. However, immediate drug treatment may completely prevent parasite growth, thus impeding the development of immunity.

To address these issues, we investigated the use of delayed death antimalaria drugs as opposed to fast-acting drugs (e.g., atovaquone-proguanil, chloroquine), which were used previously. Delayed death is a phenomenon observed in apicomplexan parasites, such as *Plasmodium* and *Toxoplasma* spp., wherein the effects of drug treatment are observed in the progeny of treated parasites (44–47). This phenotype is attributed to drug-mediated inhibition of the housekeeping functions of the apicoplast, such as DNA replication, transcription, or protein translation. Delayed death-causing drugs, such as doxycycline and the longer-acting drug azithromycin, are widely used in malaria treatment and prophylaxis (48). The use of azithromycin in conjunction with the administration of sporozoites was reported to induce strong protection against homologous challenge, but blood-stage immunity was not induced, presumably because so few parasites entered the circulation from the liver (49, 50). However, delayed death drugs have never previously been used as a strategy to induce immunity following a deliberate blood-stage infection.

We report here that CII with doxycycline administered daily allows simultaneous infection and drug treatment, enabling parasite persistence, which then induces protective immune responses. We show that the persistence of infection is critical to the induction of immunity. Furthermore, CII with different parasite species in different strains of mice can induce multiple combinations of protective immune responses, resulting in immunity against homologous and heterologous parasites. CII with Plasmodium falciparum and doxycycline in human volunteers resulted in the induction of responses similar to those that protected mice from different rodent malaria parasite species. Finally, we demonstrate the ability to induce strong CII after the administration of a single dose of azithromycin at the initiation of infection.

## RESULTS

### CII with different parasite species and doxycycline chemoprophylaxis protects against lethal homologous and heterologous challenge.

**FIG 1:**
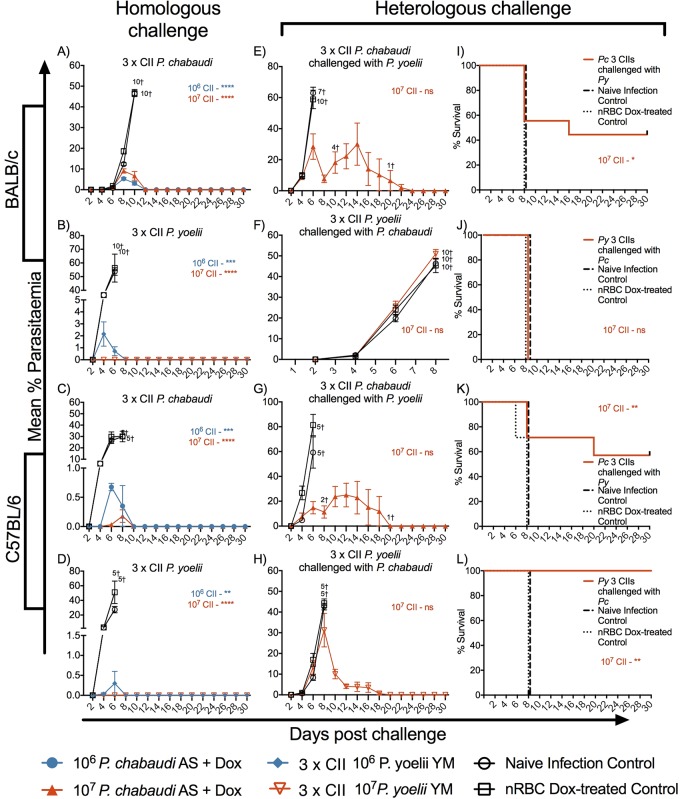
Protection after homologous and heterologous challenge of mice receiving 3 CIIs with P. chabaudi (*Pc*) or P. yoelii (*Py*). BALB/c mice (*n* = 10/group) or C57BL/6 mice (*n* = 5 to 7/group) were administered 10^6^ or 10^7^ P. chabaudi (A, C, E, G) or P. yoelii (B, D, F, H) parasites with doxycycline (Dox) treatment (50 mg/kg) over a 7-day period; age-matched control mice received 10^7^ nRBCs (nRBC and doxycycline treated) or no treatment (naive infection). Mice were rested for 2 weeks between CIIs. At 4 weeks after the last day of treatment, the mice were challenged with 10^5^ homologous (A to D) or heterologous (E to H) parasites. Parasitemia was monitored every 2nd day postchallenge. The data are expressed as the mean ± SEM. †, number of mice that were euthanized. The peak parasitemia between immunized and control mice was compared by an unpaired *t* test. (H to K) Survival curves for mice undergoing heterologous challenge. Significant differences in survival between immunized and nRBC- and doxycycline-treated mice were determined using the Gehan-Breslow-Wilcoxon test. *, *P* < 0.05; **, *P* < 0.01; ***, *P* < 0.001; ****, *P* < 0.0001; ns, not significant.

We first asked if mice receiving CII with doxycycline chemoprophylaxis developed protection against homologous or heterologous challenge. To achieve this, our studies were conducted with two rodent malaria parasites (P. chabaudi AS and P. yoelii YM) in two mouse strains (BALB/c and C57BL/6), as different parasite and mouse strain combinations can induce various mechanisms of immunity and elicit different disease manifestations (51). Mice were assessed after CII with 10^6^ or 10^7^ parasites per immunization given once or thrice. Control mice received normal red blood cells (nRBCs) and doxycycline. Low levels of parasite DNA were detected for up to 1 week after the administration of each inoculum of parasites (see Fig. S1 in the supplemental material). At 4 weeks after the last CII, mice were challenged, and we observed that all regimens induced protection against homologous parasites (Fig. 1A; see also Fig. S2 and S3 in the supplemental material), with three CII cycles with 10^7^ parasites being optimal. Heterologous immunity to a different *Plasmodium* species was also observed in both mouse strains receiving P. chabaudi CII and C57BL/6 mice given P. yoelii CII; this protection was not as strong as that against the homologous species (Fig. 1; see also Fig. S4 in the supplemental material). Peak parasitemia following heterologous challenge was significantly different only between C57BL/6 mice receiving P. chabaudi and the control groups (*P* < 0.01); however, survival was significant in all immunized C57BL/6 mice (*P* < 0.01) and BALB/c mice receiving P. chabaudi CII (*P* < 0.05).

**FIG 2:**
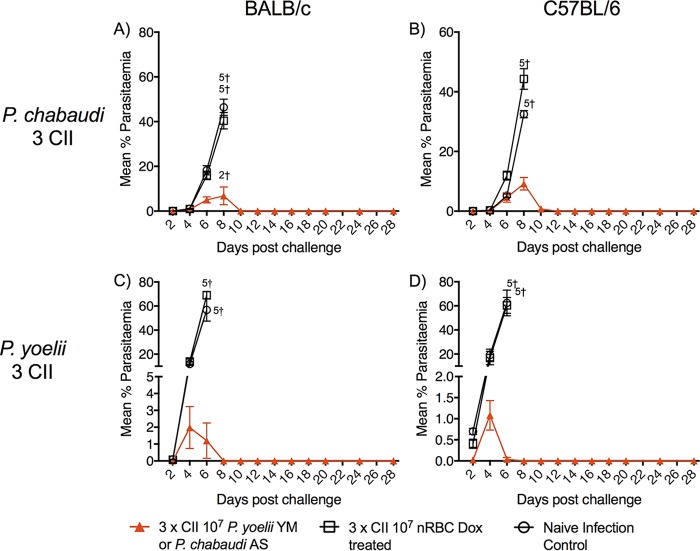
CII-induced protection at 3 months. BALB/c and C57BL/6 mice (*n* = 5/group) receiving 3 CIIs with 10^7^ P. chabaudi (A, B) or P. yoelii (C, D) parasites were rested for 3 months after the last CII before receiving a homologous challenge. Protection was assessed by monitoring of parasitemia by the use of a blood film every 2nd day postchallenge. The data represent the mean ± SEM. †, number of mice that were euthanized.

To assess if immunity was long-lived, BALB/c and C57BL/6 mice were rested for 3 months after receiving the last of three CIIs (P. chabaudi or P. yoelii) before receiving a homologous challenge. All immunized mice displayed strong antiparasitic immunity against challenge (Fig. 2), with peak parasitemias being significantly different from the peak parasitemia in control mice (*P* < 0.0001); however, two BALB/c mice receiving 3 CIIs with P. chabaudi died at a low parasitemia.

### CII with a delayed death antimalaria drug extends parasite exposure.

**FIG 3:**
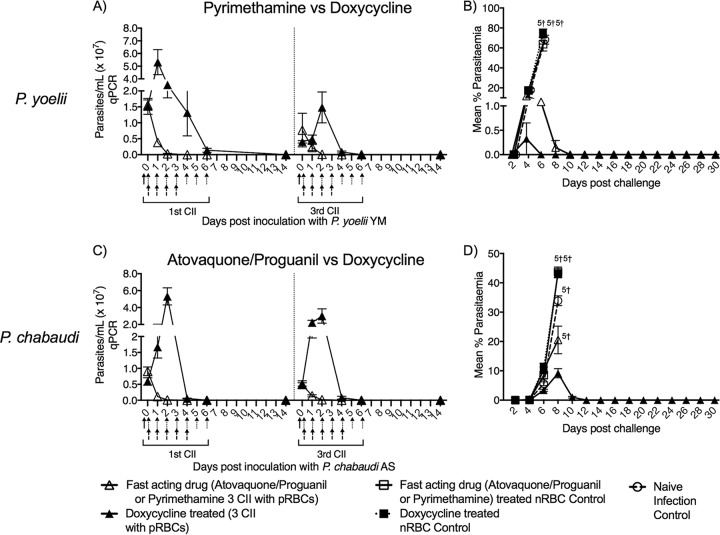
Persistence of malaria parasites under chemoprophylaxis with a fast-acting or delayed death drug. (A and C) Parasite density was assessed in blood from mice (*n* = 5/group) receiving 10^7^ pRBCs of P. yoelii with pyrimethamine treatment or P. chabaudi with atovaquone-proguanil treatment. An age-matched cohort of mice received a similar infection and treatment with doxycycline. Solid arrow, time of inoculation; dotted arrow, doxycycline administration; dashed arrow, atovaquone-proguanil or pyrimethamine administration. (B and D) Mice received a homologous challenge 4 weeks after the last CII, and parasitemia was assessed by the use of blood films. The data are expressed as the mean ± SEM. †, number of mice that were euthanized.

We asked whether CII with doxycycline elicited protection due to parasite persistence beyond one cycle. To assess this, we compared persistence and protection against homologous challenge in mice receiving CIIs with doxycycline or a fast-acting antimalaria drug. BALB/c mice were given three CIIs with P. chabaudi or P. yoelii, and treatment with doxycycline, atovaquone-proguanil, or pyrimethamine commenced on the same day as the day of infection. Parasite persistence was monitored during the first and third CIIs by quantitative PCR (qPCR). Parasite DNA persisted for at least 1 week in mice treated with doxycycline, with complete clearance by day 14 (Fig. 3A and C). The parasite density was observed to increase initially before declining. However, mice receiving pyrimethamine or atovaquone-proguanil had an immediate decline in parasite levels, and parasites were undetectable by day 2.

Following challenge, we observed that mice that received doxycycline were protected significantly better than those receiving a fast-acting drug (Fig. 3B and D). Mice that received three P. yoelii CIIs with pyrimethamine treatment developed antiparasite immunity with a peak parasitemia of 11%, while doxycycline-treated mice had a significantly lower peak parasitemia (<1%) (*P* < 0.01) (Fig. 3B). Mice that received three P. chabaudi CIIs with atovaquone-proguanil treatment developed a lower parasitemia than control mice (*P* < 0.01; Fig. 3D); however, they all succumbed to disease, while all mice receiving doxycycline CII survived, with the peak parasitemia being 9%.

### Cellular immune responses are induced after CII.

**FIG 4:**
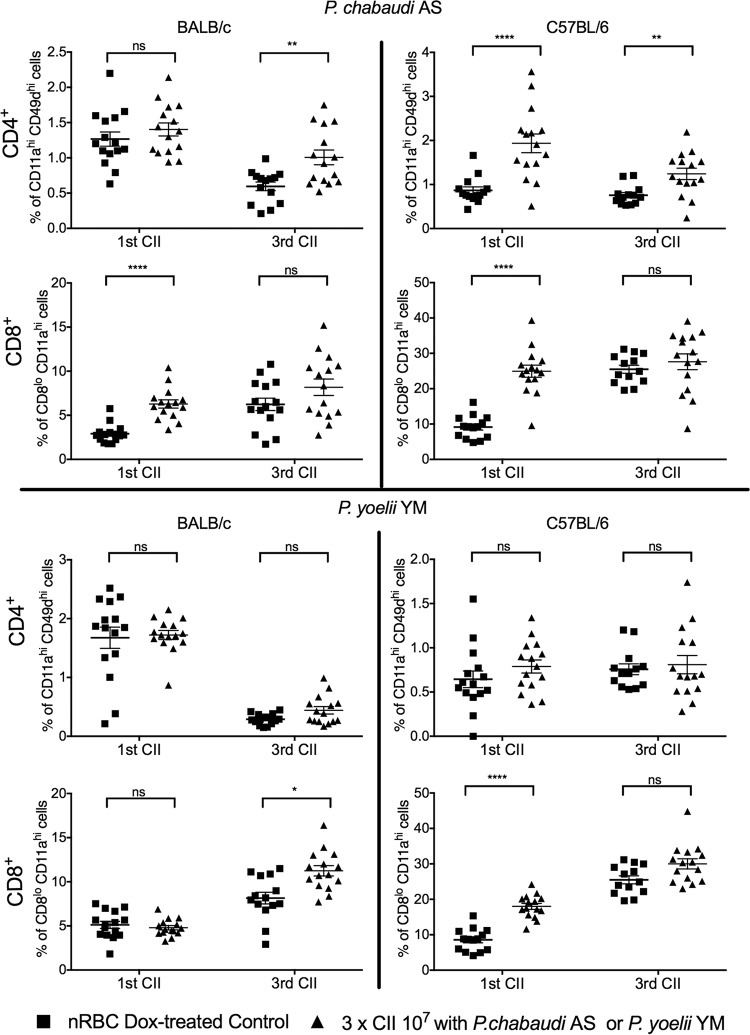
T cell activation after CII administration. Blood was collected from mice (*n* = 15/group) on day 7 postinfection with the 1st and 3rd CIIs. Mice received CIIs with P. chabaudi, P. yoelii, or nRBCs under doxycycline treatment. Activated CD4^+^ T cells were identified as CD11a^+^ CD49d^+^ cells, and activated CD8^+^ T cells were identified as CD8^lo^ CD11a^hi^ cells. The data represent the mean ± SEM and were analyzed by two-way ANOVA followed by Tukey’s multiple-comparison test. *, *P* < 0.05; **, *P* < 0.01; ****, *P* < 0.0001; ns, not significant.

We next asked if cellular immunity played a significant role in CII-induced protection. Early activation of CD4^+^ and CD8^+^ T cells was initially assessed by measuring the expression of CD11a and CD49d (52, 53) in peripheral blood T cells at 7 days postinfection for the first and third CIIs (Fig. 4). We observed that after the first CII with P. chabaudi, CD8^+^ T cells were predominantly activated in both BALB/c and C57BL/6 mice (*P* < 0.0001); however, after the 3rd CII, CD4^+^ T cells but not CD8^+^ T cells were significantly activated compared to the level of activation in control mice receiving equivalent numbers of nRBCs and doxycycline treatment (*P* < 0.01). In the P. yoelii model, there were no significant differences in CD4^+^ T cell activation after the 1st or 3rd CII in both strains. Activation of CD8^+^ T cells was observed in C57BL/6 mice after the 1st CII (*P* < 0.001), while in BALB/c mice, CD8^+^ T cell activation was observed after the 3rd CII (*P* < 0.05). Thus, different patterns of T cell activation were observed across the different parasite-mouse combinations.

**FIG 5:**
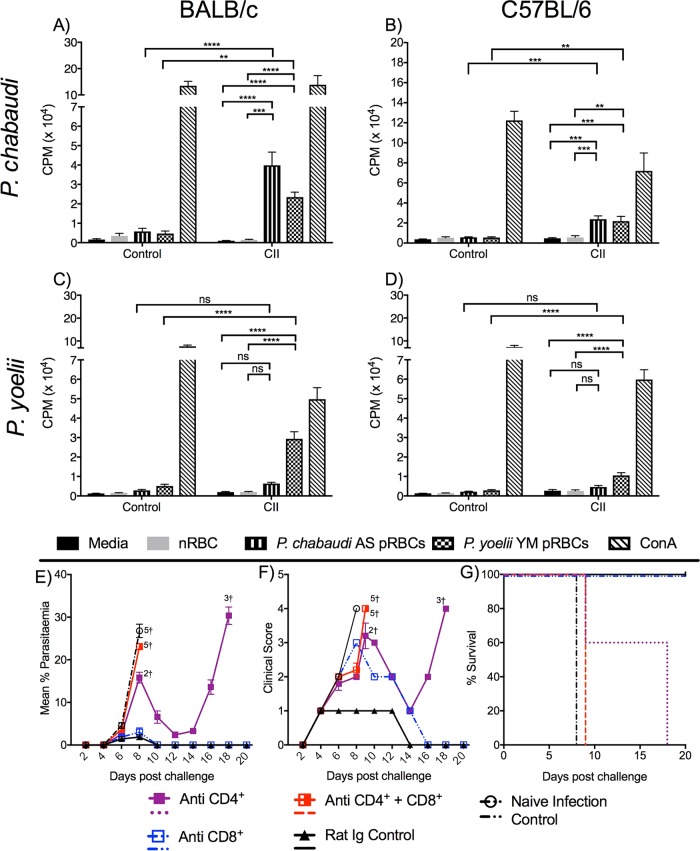
CII-induced cell-mediated immunity. The proliferation of splenocytes in response to homologous and heterologous pRBCs was investigated in BALB/c and C57BL/6 mice (*n* = 3/group) that had received 3 CIIs with 10^7^ P. chabaudi (A, B) or P. yoelii (C, D) parasites; control mice received equivalent nRBCs with drug treatment. Lymphocyte proliferation was estimated by the incorporation of [^3^H]thymidine and measured as the number of counts per minute (CPM). Samples were tested in triplicate. The data represent the mean ± SEM and were analyzed by two-way ANOVA followed by Tukey’s multiple-comparison test. **, *P* < 0.01; ***, *P* < 0.001; ****, *P* < 0.0001; ns, not significant. The role of CD4^+^ and CD8^+^ T cell subsets in P. chabaudi CII-induced protection in BALB/c mice (*n* = 5/group) was further investigated by immune depletion of one or both T cell subsets on days −2, −1, 4, and 8 relative to the time of homologous challenge on day 0. (E and F) Protection was assessed by determination of the level of parasitemia by the use of a blood film (E) and the clinical score (F). (G) Percentage of mice surviving the challenge.

Spleen cell proliferative responses to homologous and heterologous parasites were assessed for all mouse-parasite combinations at 4 weeks after the 3rd CII. Again, different responses were observed depending on the mouse strain and parasite species. Cells from BALB/c (Fig. 5A) and C57BL/6 (Fig. 5B) mice immunized with P. chabaudi proliferated in response to homologous and heterologous parasites; however, cells from both strains immunized with P. yoelii proliferated in response to homologous but not heterologous parasites (Fig. 5C and D). These findings are consistent with our observations of protection, or a lack thereof, during challenge.

Given the significant spleen cell proliferative responses to both homologous and heterologous antigens in the P. chabaudi model, we next determined whether protection was mediated by effector T cells. BALB/c mice immunized by P. chabaudi CII were depleted of CD4^+^ and CD8^+^ T cell subsets using monoclonal antibodies (Fig. 5E and F). This resulted in the depletion of 99.5% of CD4^+^ T cells and 91.5% of CD8^+^ T cells. Mice depleted of both CD4^+^ and CD8^+^ T cells were unable to control infection and showed signs of severe disease, similar to the findings for the naive infection controls (*P* > 0.05). Conversely, mice depleted of only CD8^+^ T cells controlled infection similarly to vaccinated mice receiving normal rat Ig (*P* > 0.05). Mice depleted of only CD4^+^ T cells could partially control the parasitemia, with 60% of mice surviving at day 8; however, all mice succumbed to challenge by day 18 (Fig. 5G). These data suggest that both CD4^+^ and CD8^+^ T cells can contribute to protection.

**FIG 6:**
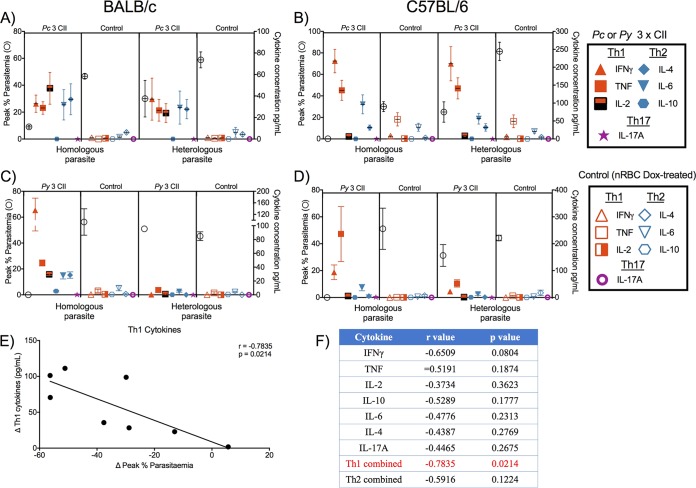
Association of peak parasitemia during challenges with cytokine production in mice receiving CII. (A to D) BALB/c and C57BL/6 mice received 3 CIIs with 10^7^ P. chabaudi or P. yoelii parasites; control mice received an equivalent number of nRBCs with doxycycline treatment. At 4 weeks after the last CII, mice received either a homologous or a heterologous challenge (*n* = 5 to 10/group). The average peak parasitemia during these challenges is plotted against the left *y* axis. Cytokine production was measured in the supernatants from homologous or heterologous parasite-stimulated splenocytes (the cytokine concentration is plotted against the right *y* axis). (E) Correlation of Th1 cytokine (IFN-γ, TNF, IL-2) production to peak parasitemia in mice receiving P. chabaudi/P. yoelii CII during homologous or heterologous challenges. The background value (that for nRBC- and doxycycline-treated mice) was subtracted from the values for the parasite-stimulated/immunized groups (change in Th1 cytokine levels [ΔTh1 cytokines]/change in peak percent parasitemia [ΔPeak percent parasitemia]). (F) Correlation statistics for individual or combined Th1/Th2 cytokines. *r*, Pearson’s correlation coefficient.

Using the supernatant from stimulated splenocytes, we found significantly increased Th1 cytokine responses (IFN-γ, IL-2, TNF) and IL-10 responses in all parasite-rodent CII combinations (see Tables S1 and S2 in the supplemental material). BALB/c mice immunized by P. yoelii CII showed increased Th2 responses across all cytokines (IL-6, IL-4, and IL-10); all other models had a significant IL-10 or IL-6 response, or both. As with the splenocyte proliferative responses, significant cytokine responses to homologous parasites were observed both in mice immunized by P. chabaudi CII and in mice immunized by P. yoelii CII; however, significant cytokine responses to heterologous parasites were observed only in mice immunized by P. chabaudi CII. Increased production of cytokines, particularly IFN-γ and TNF, correlated with low peak parasitemias during homologous or heterologous challenges (Fig. 6A to D). Examining the correlation between cytokine levels and control of infection, we observed a significant inverse relationship between peak parasitemia and a broad Th1 cytokine response (comprising parasite-induced IFN-γ, TNF, and IL-2) when comparing all combinations of mouse strains that were challenged and the different species of parasites (homologous and heterologous) (*r* = −0.7835; *P* = 0.0214; Fig. 6E). When looking at correlations with individual cytokine responses, no correlations reached significance, but the association with IFN-γ was the strongest (Fig. 6F).

### Humoral responses play a significant role in P. yoelii CII-induced protection.

**FIG 7:**
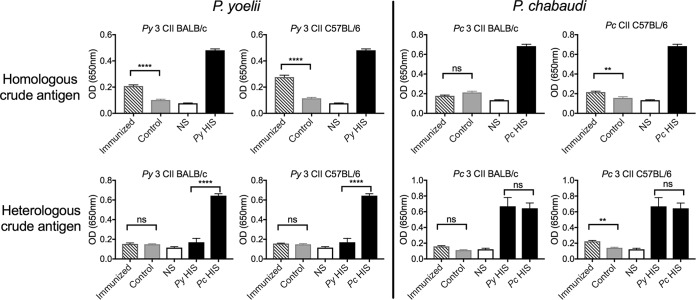
Parasite-specific antibody responses to homologous and heterologous antigens in mice receiving CII. Prechallenge serum was collected from control (nRBC- and doxycycline-treated) mice and BALB/c and C57BL/6 mice (*n* = 10/group) receiving P. chabaudi or P. yoelii CII. The production of total IgG to crude homologous or heterologous whole-parasite antigens was measured by ELISA; serum was diluted at 1:200. Serum from naive (NS) and hyperimmune (HIS) mice was included as a negative and a positive control, respectively. Results are expressed as the optical density (OD) at 650 nm. Data represent the mean ± SEM and were analyzed using one-way ANOVA followed by Tukey’s multiple-comparison test. **, *P* < 0.01; ****, *P* < 0.0001; ns, not significant.

**FIG 8:**
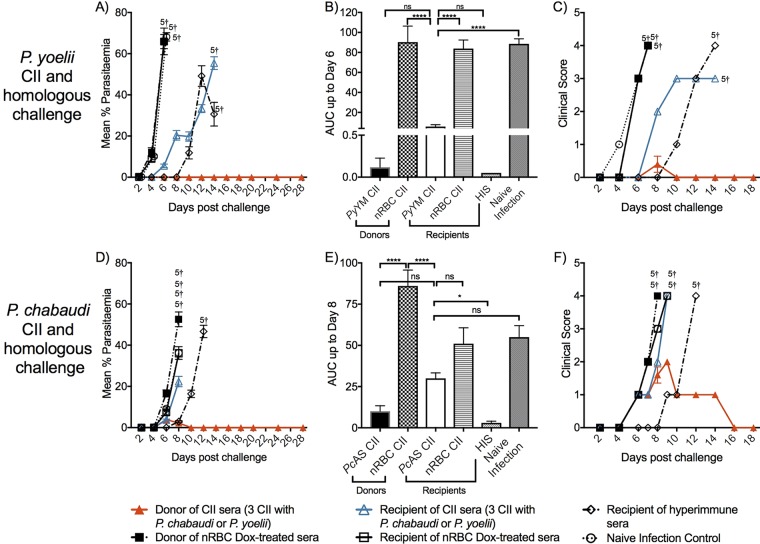
Passive transfer of pooled sera. Serum was collected from BALB/c mice immunized with 3 CIIs of P. yoelii YM (*Py*YM) or P. chabaudi AS (*Pc*AS). Serum was additionally collected from nRBC- and doxycycline-treated mice and hyperimmune control mice (HIS). Naive BALB/c mice (*n* = 5) each received 500 μl of pooled serum on days −1, 0, and 1 relative to the time of homologous challenge on day 0. Immunized donor and naive mice (*n* = 5/group) were additionally challenged and acted as controls. Mice were monitored for parasitemia (A and D) and the clinical score (C and F). The area under the curve (AUC) was calculated based upon the parasitemia curves up to the indicated days (B and E). †, number of mice that were euthanized. The data represent the mean ± SEM and were analyzed using one-way ANOVA followed by Tukey’s multiple-comparison test. *, *P* < 0.05; ****, *P* < 0.0001; ns, not significant.

We then asked whether there were correlates of protection involving antibody responses. Following CII, specific antibodies to homologous antigen were not detectable in serum from BALB/c mice receiving P. chabaudi CII, nor were they detectable in 3 of the 4 CII models when tested against heterologous parasites (Fig. 7). To gauge the function of the antibodies to homologous parasites, we tested whether serum from immunized BALB/c mice could adoptively transfer protection to naive recipient mice and observed that those receiving serum from mice that had received P. yoelii CII but not those receiving serum from mice that had received P. chabaudi CII had a delayed onset of parasitemia and clinical scores in comparison to the control groups (Fig. 8); analysis of the area under the curve (AUC) (Fig. 8B and E) found that mice receiving serum from BALB/c mice receiving P. yoelii CII were not significantly different from that in the group receiving hyperimmune serum (HIS) (*P* > 0.05) generated following multiple infections and drug cure, indicating the presence of functional antibodies. Therefore, a significant component of immunity to P. yoelii, but not P. chabaudi, could be attributed to antibody.

**FIG 9:**
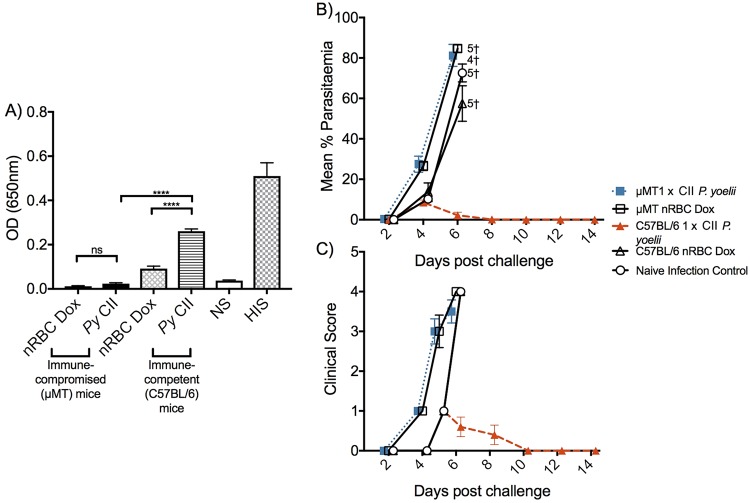
Role of B cells in mice receiving P. yoelii CII. Humoral immunity was assessed in μMT and C57BL/6 mice (*n* = 4 or 5/group) receiving one P. yoelii CII. (A) Prior to challenge, samples were taken from mice and analyzed for P. yoelii-specific antibodies by ELISA; the serum was diluted at 1:200. Serum from naive (NS) and hyperimmune (HIS) mice was included as a negative and a positive control, respectively. Results are expressed as the optical density (OD) at 650 nm. (B, C) Mice received a homologous challenge with 10^5^ P. yoelii parasites and were monitored by assessment of the level of parasitemia by the use of a blood film (B) and the clinical score (C). The data represent the mean ± SEM. †, number of mice that were euthanized. Data were analyzed by two-way ANOVA followed by Tukey’s multiple-comparison test. ****, *P* < 0.0001; ns, not significant.

To confirm whether there was a role of B cells and antibody in mice receiving P. yoelii CII, B cell-deficient (μMT) mice (on a C57BL/6 mouse background) were given one CII and challenged 4 weeks later alongside C57BL/6 mice receiving the same treatment (Fig. 9). We were not able to acquire B cell-deficient BALB/c mice. Prior to challenge, serum samples were obtained and analyzed by enzyme-linked immunosorbent assay (ELISA) for the presence of parasite-specific antibodies. Immunized μMT mice had no P. yoelii-specific antibodies, while immunized C57BL/6 mice had significantly higher levels of parasite-specific antibodies than control mice (*P* < 0.0001; Fig. 9A). Immunized μMT mice were unable to control infection, whereas immunized C57BL/6 mice could control infection and disease, with a peak parasitemia of 9% occurring on day 4 (Fig. 9B and C). Collectively, the data from adoptive transfer studies and from the study of μMT mice suggest that antibody contributes to protection in mice receiving P. yoelii CII but not in BALB/c mice receiving P. chabaudi CII. We did not analyze a role for protection against P. chabaudi in C57BL/6 mice.

### Pilot clinical study of CII with doxycycline.

The rodent data demonstrated different patterns of immune induction following CII with doxycycline, but with parasite-induced inflammatory responses showing a constant association with protection and with a variable role for antibody. We thus asked whether humans might also mount responses to P. falciparum CII with doxycycline consistent with one or more of the rodent responses. We investigated the persistence and immunogenicity of purified P. falciparum 7G8-parasitized red blood cells (pRBCs) administered with doxycycline treatment in volunteers. Four malaria-naive male volunteers (participant 1 [P1] to P4) were recruited. They were healthy, were of Caucasian descent, and had a mean age of 23 years. Study participants were infected with pRBCs derived from a cryopreserved cultured blood-stage malaria parasite-infected cell bank previously described (54). Parasitized RBCs from this cell bank were thawed and cultured *in vitro*. Trophozoite-stage parasites were harvested and purified, with 3 × 10^6^ magnet-purified trophozoites being administered intravenously to each volunteer. Volunteers received one CII treatment, with P1 and P2 being treated initially and then P3 and P4 being treated. Doxycycline treatment (100 mg/day for 21 days) commenced 1 h after administration of parasites. Adverse events deemed possibly related to the administration of the inoculum were noted in 3 of the 4 participants (Table S3). All were of mild severity.

### Persistence of P. falciparum during doxycycline treatment.

**FIG 10:**
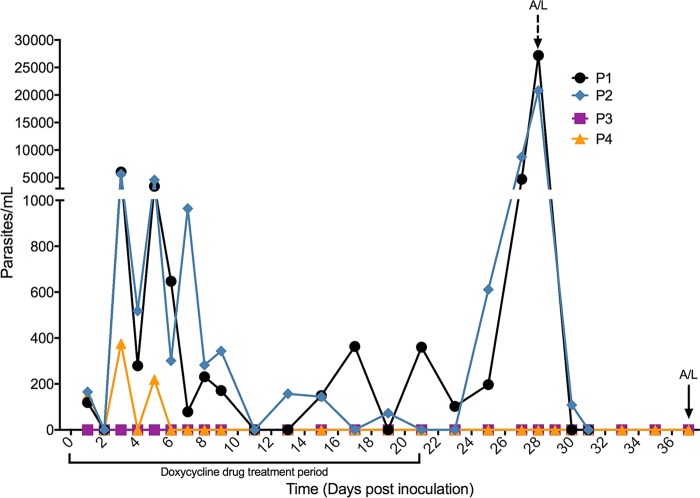
Persistence of P. falciparum during CII in individual participants. Participants (P1 to P4) were subjected to one inoculation with 3 × 10^6^ P. falciparum 7G8 parasites and doxycycline treatment (one 100-mg tablet daily), with P1 and P2 receiving their inoculation on a different day than P3 and P4. Doxycycline treatment was administered approximately an hour after inoculation (day 0) and was continued daily for 21 days. To assess the parasite density during CIIs, blood was taken and processed for qPCR analysis. Dashed arrow, the day that P1 and P2 were given artemether-lumefantrine (A/L); solid arrow, the day that P3 and P4 received A/L treatment.

None of the volunteers developed a microscopically detectable infection. Parasite densities were monitored and measured using qPCR (Fig. 10). Following pRBC inoculation and initiation of doxycycline treatment, all participants bar one (P3) developed infection detectable by qPCR during the study. Parasite densities in P1, P2, and P4 initially increased from day 1 (119 to 165 parasites/ml) and peaked on day 3 (376 to 6,020 parasites/ml), subsequently decreasing thereafter. Infection in P4 was detectable for 2 cycles, before it became undetectable by day 5 and remained as such until the end of the study. Parasite density also declined in P1 and P2; however, infection was not completely eradicated by drug treatment. This resulted in a persistent low level of parasites that appeared to be controlled by doxycycline treatment. The parasitemia in these participants increased after the cessation of drug treatment, and volunteers P1 and P2 required rescue treatment on day 28 with artemether-lumefantrine (A/L). Participants 3 and 4 underwent precautionary rescue treatment with A/L on day 37, in accordance with the study protocol.

### P. falciparum CII-induced cellular responses.

**FIG 11:**
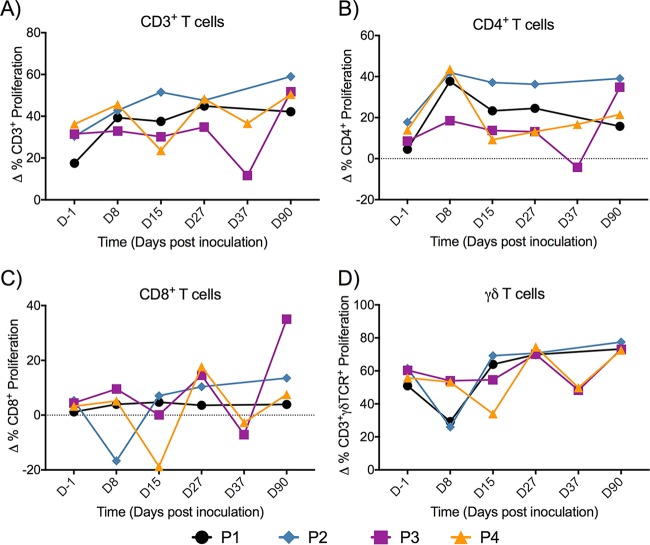
Parasite-specific proliferative responses in human T cell subsets. Isolated PBMCs collected before (day −1) and after CII commencement (days 8, 15, 27, 37, and 90) were thawed and stimulated with uninfected RBCs and purified P. falciparum 7G8 trophozoite-parasitized RBCs (pRBCs) for 7 days. Prior to stimulation, cells were stained with violet proliferation dye (VPD) and after 7 days were stained for analysis of specific T cell populations: CD3^+^ (A), CD4^+^ (B), CD8^+^ (C), and γδ T (D) cells. ΔCD3^+^/CD4^+^/CD8^+^/γδ T cell proliferation refers to the pRBC-stimulated counts with the background counts (those for PBMCs stimulated with uninfected RBCs) subtracted. D, day.

Peripheral blood mononuclear cells (PBMCs) were assessed for the responsiveness to P. falciparum 7G8 *in vitro* by measurement of dye dilution using flow cytometry (for estimating the proliferation of T cell subsets) (Fig. 11). All samples from a given participant were analyzed in the same assay. Comparing the findings obtained on day −1 to those obtained on day 90 using a paired *t* test, we observed an increase in total CD3^+^ (*P* < 0.01), CD4^+^ (*P* < 0.05), and γδ T (*P* < 0.01) cell proliferation in all volunteers and increased CD8^+^ (*P* > 0.05) T cell responses in P3 (Fig. 11). The proliferative responses of all cell subsets fluctuated over the 3-month sampling period.

**FIG 12:**
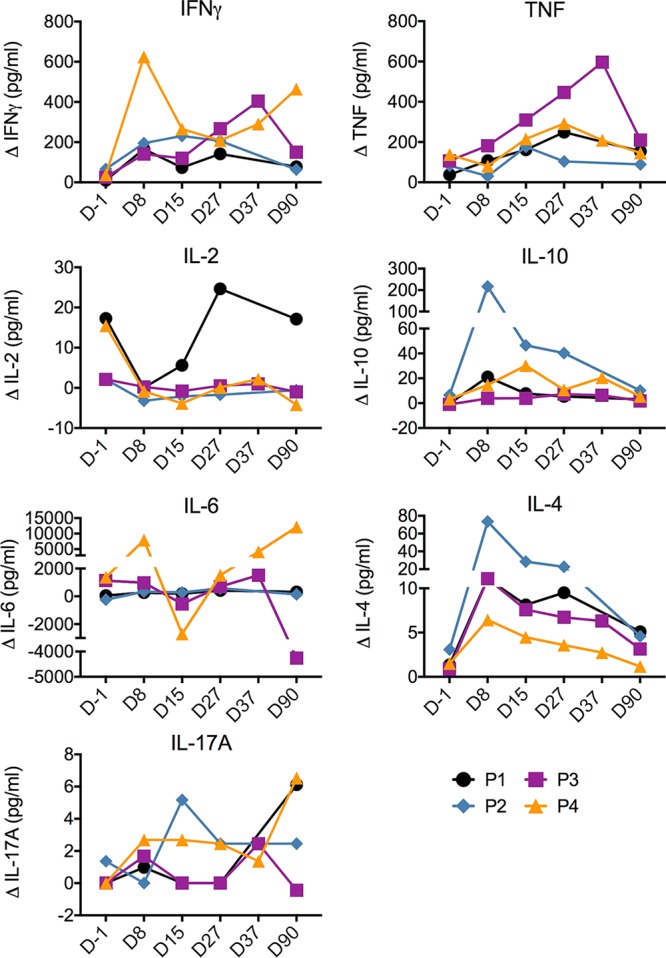
P. falciparum-elicited cytokine responses. Culture supernatants from parasite-stimulated lymphocytes were collected, with the supernatants from triplicate wells being pooled, and used to quantify the levels of cytokines produced after stimulation with uninfected RBCs or P. falciparum 7G8 pRBCs. The background result (that for uninfected RBCs) was subtracted from the results for the pRBC-stimulated samples.

Analysis of secreted cytokines in the cell culture supernatants after 6 days showed elevated levels of parasite-specific IFN-γ and TNF responses compared to those preinoculation in all volunteers (Fig. 12). The other cytokines tested (IL-2, IL-10, IL-6, IL-4, and IL-17A) were detected in different individuals but not in a consistent manner between participants or over time.

### Antibody responses induced by P. falciparum CII.

**FIG 13:**
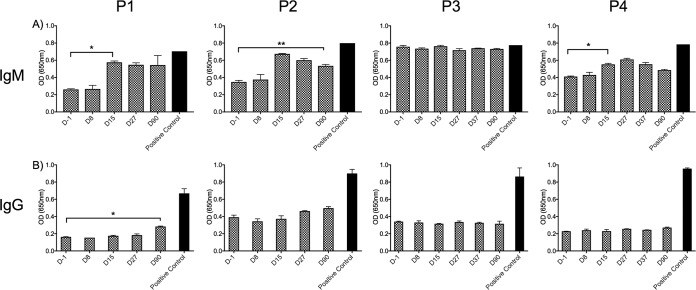
Parasite-specific IgM and total IgG responses in volunteers. Participants (P1 to P4) received one cycle of CII. Pre- and postinoculation plasma samples were collected from the participants at several time points during the study (days −1, 8, 15, 27, 37, and 90). The production of IgM (A) and total IgG (B) in response to crude P. falciparum 7G8 whole-parasite antigen was measured by ELISA; samples were diluted at 1:50. Serum from a malaria-experienced individual was included as a positive control. Results are expressed as the optical density (OD) at 650 nm. Samples were run in duplicate. Data represent the mean ± SEM. Groups were analyzed by repeated measures one-way ANOVA followed by Dunnett’s multiple-comparison test, comparing pre- to postinoculation time points. *, *P* < 0.05; **, *P* < 0.01.

Plasma samples which had been collected from the volunteers prior to inoculation and at several time points postinoculation were assessed for the presence of IgM and total IgG against P. falciparum 7G8 crude antigen (Fig. 13A and B). Following inoculation, P1, P2, and P4 developed IgM antibodies that on day 15 or 90 were present at levels significantly higher than the preinoculation (day −1) levels (*P* < 0.05). For P3, there were no significant differences in IgM levels when those at the pre- and postinoculation time points were compared (*P* > 0.05). With the exception of P1, there were no significant differences in parasite-specific IgG levels postinoculation and preinoculation (day −1) (Fig. 13B). IgG levels in P1 were found to be significantly different from those on day −1 only on day 90 (*P* < 0.05).

### Infection and one dose of a delayed death antimalaria drug can elicit protection from lethal infection.

The ability of all four human volunteers to respond to a single CII by producing two cytokines that have been associated with malaria immunity (IFN-γ and TNF) and the ability of three of the four individuals to produce parasite-specific antibodies demonstrated a strong similarity with the mouse CII data and suggested that CII may represent a strategy to induce protective immunity in humans. However, a protocol that requires daily treatments with doxycycline is not a viable strategy. We thus sought a methodology that might control parasite growth with a single drug treatment.

**FIG 14:**
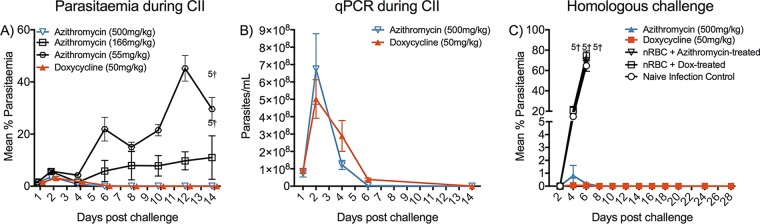
Single-dose azithromycin administration in one P. yoelii CII. BALB/c mice (*n* = 5/group) were administered azithromycin (55 mg/kg, 155 mg/kg, or 500 mg/kg) by subcutaneous injection (100 μl) in conjunction with infection with 10^7^ P. yoelii parasites. A control group was administered the doxycycline regimen described in the text. (A) Parasitemia was assessed during the CII across all groups by the use of blood films. (B) Additionally, blood was collected from mice receiving azithromycin (500 mg/kg) or doxycycline (50 mg/kg) to assess the parasite density in mice by qPCR during immunization. (C) After 4 weeks, groups receiving azithromycin (500 mg/kg) or doxycycline (50 mg/kg) were subjected to homologous challenge, and protection was monitored by determination of the level of parasitemia using blood films.

For this, we tested azithromycin, as it has a comparatively longer half-life than doxycycline (7 h versus 2.4 h in mice [55–58] or 65 to 68 h versus 15 to 25 h in humans [59–61]). Azithromycin additionally has a better safety profile in pregnant women and may be used by children. We tested azithromycin using one CII with P. yoelii. Mice received P. yoelii infection under the 7-day doxycycline protocol (50 mg/kg of body weight/day) or with various doses of azithromycin (55 mg/kg, 166 mg/kg, and 500 mg/kg), which was, however, administered as a single 100-μl subcutaneous injection. Mice receiving lower doses of azithromycin were unable to cure infection and were euthanized by day 14 (Fig. 14A); however, mice that received azithromycin at a single dose of 500 mg/kg controlled the parasite density (Fig. 14A and B) to a level similar to that for mice that underwent daily doxycycline treatment. No obvious adverse events in response to azithromycin administration were observed in mice during the immunization phase. Following subsequent challenge 4 weeks after CII, the azithromycin group was shown to be strongly protected, similar to the findings for doxycycline-treated mice (*P* < 0.05; Fig. 14C). Drug-treated controls developed parasitemia similar to the naive infection control animals, indicating that no residual azithromycin was present at the time of challenge, despite the high dose administered.

## DISCUSSION

Here, we report the novel use of delayed death antimalaria drugs combined with simultaneous blood-stage infection as an immunization approach. To our knowledge, delayed death drugs have not been used in a strategy to induce immunity following blood-stage infection. To investigate CII with doxycycline, we used two different parasite species, P. chabaudi AS and P. yoelii YM, in two different mouse strains, BALB/c and C57BL/6 mice. These species from rodents have characteristics similar to those of the human malaria parasites P. falciparum and P. vivax, respectively, and were tested in two mouse strains differing in both major histocompatibility complex type and background genes. Furthermore, the two strains exhibit a predominant Th1 or Th2 bias (51, 62–64). The use of different combinations of parasite species and mouse strains provides a robust test for the utility of the CII strategy.

In using various parasite-host combinations, we show that CIIs can elicit both cellular and humoral immune system-mediated immune responses (summarized in Table 1) with the ability to provide robust protection in immunized mice. Numerous studies investigating infection, whether natural or drug controlled after a period of delay, in various rodent-parasite models similarly illustrate the induction of different, yet protective, immune responses (65–71). A recurring finding in many studies using different methodologies is an association of IFN-γ, TNF, and/or IL-2 levels in serum or *in vitro* in response to parasite stimulation and protection (10, 26, 27, 29, 30, 37, 40, 41). Here, with a systematic study of different parasites and hosts, we demonstrate a strong association between protection and the cumulative inflammatory cytokine response following CII with delayed death drugs. Cytokine analyses also revealed an IL-10 response across all models in response to homologous pRBCs. Previous studies have shown a role of IL-10 in limiting host pathology (72, 73), although its suppressive qualities may also delay pathogen clearance (35).

**TABLE 1:** Summary of immune responses to CII with different parasite and host combinations

Immune aspect	Host	Immune response to the following Plasmodium species:		
		*P. chabaudi*	*P. yoelii*	*P. falciparum*
Cell-mediated immunity	BALB/c mice	Early activation of CD4^+^ and CD8^+^ T cells Lymphocyte proliferation in response to homologous and heterologous antigens CD4^+^ T cells critical to immunity; some CD8^+^ T cell influence	Early activation of CD8^+^ T cells Lymphocyte proliferation in response to homologous antigen	
	C57BL/6 mice	Early activation of CD4^+^ and CD8^+^ T cells Lymphocyte proliferation in response to homologous and heterologous antigens	Early activation of CD8^+^ T cells Lymphocyte proliferation in response to homologous antigen	
	Humans			Lymphocyte proliferation in response to homologous antigen (CD4^+^ and γδ T cells)
Cytokine production	BALB/c mice	Cytokine production in response to homologous and heterologous antigens Production of all Th1 cytokines (IFN-γ, TNF, IL-2) and IL-10	Cytokine production in response to homologous antigen Production of all Th1 (IFN-γ, TNF, IL-2) and Th2 (IL-6, IL-10, IL-4) cytokines	
	C57BL/6 mice	Cytokine production in response to homologous and heterologous antigens Production of Th1 (IFN-γ, TNF) and Th2 (IL-6, IL-10) cytokines	Cytokine production in response to homologous antigen Production of all Th1 cytokines (IFN-γ, TNF, IL-2) and IL-6	
	Humans			Th1 (IFN-γ, TNF)
Antibody-mediated immunity	BALB/c mice	No IgG in response to homologous and heterologous antigens	Functional IgG in response to homologous antigen but not heterologous antigen	
	C57BL/6 mice	Some parasite-specific IgG in response to homologous and heterologous antigens	Parasite-specific IgG in response to homologous antigen but not heterologous antigen B cells critical to immunity	
	Humans			Little/no IgG Parasite-specific IgM

Immune cell depletion studies with mice receiving P. chabaudi CII showed that CD4^+^ T cells were critical for protection in this model, with CD4^+^ T cells being the likely source of both the inflammatory cytokines and IL-10 (74). This finding is consistent with reports of immunity following whole-parasite vaccination being CD4^+^ T cell dependent in rodents (75, 76). CD4^+^ T cell depletion, however, initially did not completely abrogate protection, suggesting that CD8^+^ T cells may also play a role in protection; the involvement of CD8^+^ T cells in blood-stage immunity has been reported in certain mouse-parasite combinations (65, 77–79).

The role for antibodies was variable. In contrast to P. chabaudi CIIs, humoral immunity was important during P. yoelii CII, as evidenced by the presence of functional parasite-specific antibodies in immunocompetent mice and a lack of protection in B cell-deficient mice. This finding is supported by previous studies which observed that the control of P. yoelii infection is B cell dependent, whereas P. chabaudi infections could resolve infection independently of B cells (69, 80), and further highlights the importance of understanding the interplay of the immune system with individual *Plasmodium* species. We noted that the amount of antibody was low in all CII models and significantly less than that for the positive controls (hyperimmune serum). While it might seem surprising that low levels of antibodies could be protective, it is worth noting that small amounts of immunoglobulin from malaria-immune adults are able to significantly reduce parasitemia when transferred into parasitemic children (43).

We attribute the protective efficacy of CII to doxycycline’s ability to allow parasites to persist for an extended period; this was demonstrated when doxycycline-treated mice exhibited longer parasite persistence during CII and better protection against challenge than mice receiving CIIs with fast-acting antimalaria drugs (atovaquone-proguanil or pyrimethamine). The parasite density under doxycycline treatment, unlike that under atovaquone-proguanil or pyrimethamine treatment, increased before drug effects were observed; this observation is in accord with the delayed death phenomenon (45). We note that detection of parasite DNA on day 6 (the last day of treatment), particularly in mice receiving P. yoelii CII, could indicate that a small subset of parasites was not successfully killed, thus suggesting subcurative treatment. This notion is further supported by the prolonged persistence of parasites in volunteers P1 and P2. In rodents, the lack of DNA detection on day 14 would therefore suggest that drug treatment had allowed enough time for the host to mount an immune response against remaining parasites. Given that this was not observed in P1 and P2, we believe that the DNA detected in mice on day 6 is likely attributed to dead parasite material.

Interestingly, we observed that mice receiving P. chabaudi CII could control heterologous infection with P. yoelii, although the inverse was not true. These findings contrast with the observations of several studies investigating cross-species immunity, which generally found that P. yoelii-immunized mice were able to resist P. chabaudi infection but not vice versa (69, 81, 82). Conversely, one study found that mice receiving primary infection with P. chabaudi were protected against lethal P. yoelii infection (66). However, these studies utilized different strains and largely investigated heterologous immunity after a self-resolving primary malaria infection.

Differences in heterologous immunity were further reflected in splenocyte proliferative responses, where mice receiving P. chabaudi CII had significant responses and concentrations of Th1 cytokines in response to heterologous antigens but mice receiving P. yoelii CII did not. The inability of heterologous antigens to induce Th1 cytokines, such as IFN-γ, therefore appears to contribute to the lack of heterologous protection. The early production of IL-12, a known inducer of IFN-γ, by antigen-presenting cells is reported to be important to innate and cell-mediated activation (83, 84), as well as antibody-mediated protection against chronic P. chabaudi infection (85). Further, P. yoelii-infected mice were observed to have increased immature dendritic cells and less IL-12 (86). Investigation of IL-12 production during P. chabaudi and P. yoelii CII may further elucidate the discrepancies in heterologous immunity observed.

The level of protection against a heterologous species was not as strong as that against the homologous species. It is expected that much stronger protection against heterologous strains of the same species would be observed. However, the protection that is observed is likely due to the high degree of conservation between target antigens of T cells (13). The almost uniform lack of cross recognition of antibodies to heterologous species (Fig. 7) is not surprising, given that antibodies often recognize polymorphic surface antigens, and further suggests that the cross-species protection observed is likely due solely to cellular immune mechanisms that are activated by conserved housekeeping antigens and enzymes (13, 87). However, the data do strongly suggest that optimal protection requires both a cellular response and an antibody response.

The ability to induce a long-lived protective immune response is critical for a successful malaria vaccine. In this study, we investigated immunity 3 months after CII and found that protection was still evident in all models. The induction of long-term immunity (9 to 12 months) and the boosting effect of natural infections are areas that need to be further investigated.

Our clinical study found that the administration of one CII with doxycycline chemoprophylaxis was well tolerated in volunteers; however, treatment was subcurative in 2/4 volunteers. Exposure to P. falciparum resulted in increased cellular responses across all volunteers; these responses were observed to fluctuate during the study, which may be explained in part by subpatent parasitemia in two of the volunteers. However, comparison of the findings at day 90, at which time individuals had been cleared of parasites, to those at day −1 still showed an increased level of proliferating T cells (CD3^+^, CD4^+^, and γδ). Parasite-specific CD4^+^ T cells have previously been observed in natural and experimental infection studies (26, 39, 88). We further observed significant levels of IFN-γ and TNF, as well as little or no IgG. Although the number of volunteers in this study was small, these observations are similar to our findings in the P. chabaudi CII model. Such similarities between P. chabaudi and P. falciparum may relate to parallels of a hematological nature (89, 90) and an immunological nature (reviewed in reference 62). Increased numbers of parasite-specific γδ T cells were observed in all volunteers. γδ T cells are known producers of IFN-γ and have been reported to be associated with protection in several studies (39, 70, 88, 91–93). The induction of IgM antibodies was additionally observed in 3 volunteers; similarly, recent studies have reported IgM detection after blood-stage exposure (94–97). Based on our rodent data, the prolonged persistence of P. falciparum in volunteers may have contributed to the immunogenicity of the inoculum; however, until volunteers are challenged, we cannot definitively determine if these immune responses are protective.

The need for repeated treatment is a distinct disadvantage of the standard CII model. We sought to reduce drug treatment by using azithromycin, which has a better safety profile and increased half-life compared with doxycycline (98). We show that parasite persistence and protective immunity can be achieved in mice with a single infection and drug treatment. However, neither the subcutaneous route of administration nor the dose of azithromycin used here is clinically indicated to treat general infections (99, 100). Higher doses of azithromycin may be tolerable in humans. No obvious adverse events were observed in the mice in the studies described here.

In conclusion, we present a novel approach to the current *in vivo* chemical attenuation methodology by using delayed death antimalaria drugs to prolong parasite persistence. Despite observing different mechanisms of immunity with different host-parasite combinations, mice were ultimately protected from challenge, with a broad Th1 cytokine response providing the best correlate of protection but with the need for antibody as well for optimal protection. We further show that a single CII in humans produces a similar immunogenicity. Lastly, we demonstrate the possibility of reducing the methodology to a single infection and treatment in mice. While these data are encouraging, the translation of CII to humans still represents a significant challenge. The work outlined here may inform future blood-stage CII approaches to vaccine development.

## MATERIALS AND METHODS

### Experimental animals and rodent parasites.

Four- to 6-week-old female BALB/c and C57BL/6 mice were obtained from the Animal Resource Centre (ARC; Perth, Australia). μMT mice were initially obtained from the Queensland Institute for Medical Research (QIMR; Brisbane, Australia) and bred in-house. All animals were housed in the Institute for Glycomics animal facility at Griffith University per physical containment level 2 regulations under pathogen-free conditions. Cloned lines of P. chabaudi AS and P. yoelii YM were initially obtained from Richard Carter (Edinburgh, United Kingdom) and maintained by serial passage in inbred mice.

### Immunization of mice with rodent *Plasmodium* spp. under antibiotic prophylaxis.

One round of CII was conducted as follows: P. chabaudi AS (trophozoite stage)- or P. yoelii YM (asynchronous)-infected blood was collected into lithium-heparin blood collection tubes (BD Biosciences, Franklin Lakes, NJ). Blood was washed with 1× phosphate-buffered saline (PBS), and an immunizing dose of 1 × 10^7^ pRBCs (unless stated otherwise) was prepared to be intravenously injected into mice. Control groups received 1 × 10^7^ normal RBCs (nRBCs). One hour later, mice received an intraperitoneal injection of doxycycline hyclate in PBS (50 mg/kg; Sigma-Aldrich, St. Louis, MO). Doxycycline treatment was administered daily for the next 6 days. Groups receiving a total of 3 CIIs were rested for 2 weeks between each round of CII.

Azithromycin (500 mg/kg, 166 mg/kg, or 55 mg/kg; Sigma-Aldrich), prepared at 100 mg/ml in propylene glycol (Sigma-Aldrich) (101), was administered subcutaneously an hour after P. yoelii infection.

### Challenge of mice with blood-stage parasites and monitoring of disease.

Control and immunized mice were challenged 4 weeks after the last CII with 1 × 10^5^ P. chabaudi AS or P. yoelii YM for homologous or heterologous experiments. Age-matched naive mice were challenged alongside the experimental groups to account for residual drug. Parasitemia was monitored every alternate day by the use of Giemsa-stained blood films, and the hemoglobin level was measured every 4 days (HemoCue 201^+^ analyzer; Hemocue, Angelholm, Sweden). Percent parasitemia was calculated as the number of infected RBCs divided by total RBC count (a minimum of 300 total RBCs were counted) multiplied by 100. To assess health, mice received clinical scores every 2 days (76). Mice that showed signs of severe disease were euthanized using CO_2_ gas. Observer bias was reduced through experimental blinding, except for experiments requiring immune cell depletion.

### Quantification of rodent malaria parasites in blood by qPCR.

DNA was extracted using a QIAamp DNA blood minikit (Qiagen, Victoria, Australia) per the manufacturer’s instructions, and qPCR was performed as outlined previously (75). Standards and samples were run as triplicates.

### Assessment of CD4^+^ and CD8^+^ T cell activation after CII.

T cell activation was assessed as previously described (76), with the following modifications. Samples were incubated on ice with 50 μl of Fc receptor block (clone 2.4G2; BD Biosciences) for 10 min. Cells were stained with an antibody master mix (all from BD Biosciences) containing CD3 (clone 17A2 labeled with V450), CD4 (clone RM4-5 labeled with V500), CD8 (clone 53.6.7 labeled with peridinin chlorophyll protein [PerCp]-Cy5.5), CD11a (clone 2D7 labeled with fluorescein isothiocyanate [FITC]), and CD49d (clone R1-2 labeled with phycoerythrin [PE]) in MACS buffer on ice for 20 min, before 3 washes with MACS buffer. Cells were resuspended in magnetically activated cell sorting (MACS) buffer and analyzed using an LSR Fortessa flow cytometer (FACSDiva software, v6; BD Biosciences) and FlowJo software (v10). See Fig. S5 in the supplemental material for the gating strategy.

### Splenocyte proliferation assays.

Spleens were harvested from mice and processed by lysing the RBCs with Gey’s lysis buffer. Single-cell suspensions were prepared, and cells were diluted to 4 × 10^6^ cells/ml in complete culture medium (RPMI 1640 supplemented with 1% l-glutamine [100×], 1% penicillin-streptomycin, and 0.1% 2-mercaptoethanol with 10% heat-inactivated fetal bovine serum [FBS]). Samples were dispensed at 100 μl/well in triplicate and cultured with concanavalin A (ConA; 10 μg/ml), P. chabaudi AS, and P. yoelii YM pRBCs (5 × 10^6^ pRBCs/ml), an equivalent concentration of nRBCs, or complete medium for 72 h at 37°C. Culture supernatants were removed and stored at −80°C for cytokine analysis and replaced with fresh complete medium. Cultures were pulsed with 1 μCi/well of [^3^H]thymidine (Perkin Elmer, Waltham, MA) after 54 h and left at 37°C for 18 h. Incorporation was stopped by storing the culture at −80°C, and the contents of the plates were harvested onto fiberglass mats. [^3^H]thymidine incorporation was measured on a PerkinElmer MicroBeta counter.

### Cytokine analysis.

The levels of cytokines secreted by splenocytes or participant lymphocytes were measured using supernatants removed from the assay mixtures and stored at −80°C. Mouse or human Th1/Th2/Th17 cytokine bead array (CBA) kits (BD Biosciences) were used according to the manufacturer’s instructions, with modifications for the mouse kit outlined previously being used (76). Clinical samples were not diluted for the human Th1/Th2/Th17 CBA kit. For IFN-γ analysis, samples were diluted 1:20 in assay diluent and analyzed using an IFN-γ flex set (BD Biosciences) according to the manufacturer’s instructions. Samples were analyzed using an LSR Fortessa flow cytometer and FCAP Array software (v1.0.1; BD Biosciences).

### Assessment of antibodies by ELISA.

Crude parasite antigen was prepared as previously described (75). MaxiSorp Nunc Immuno 96-well plates (Thermo Fisher, Carlsbad, CA) were coated overnight at 4°C with 5 μg/ml or 10 μg/ml of parasite antigen in bicarbonate coating buffer (pH 9.6). The plates were blocked with 10% skim milk for 1.5 h at 37°C and washed with PBS-Tween 20. Serum was diluted 2-fold in 5% skim milk, starting at 1:50 or 1:100. After 1.5 h of incubation, the plates were washed 4 times and incubated with horseradish peroxidase-conjugated total IgG (goat anti-mouse IgG [Bio-Rad, Hercules, CA], goat anti-human IgG [Abcam, Cambridge, United Kingdom]) or IgM (goat anti-human IgM; Chemicon, Tokyo, Japan). Tetramethylbenzidine (BD Biosciences) was added, and the plates were incubated at room temperature for 10 to 15 min and read at 650 nm.

### Passive antibody transfer.

Serum from immunized, control, or hyperimmune mice collected over a period of 4 weeks was pooled approximately 1 week after receiving the last CII. Hyperimmune mice were generated by administering >3 infections with P. chabaudi AS or P. yoelii YM, with treatment starting at 10% to 20%. Atovaquone-proguanil (100 μl, 0.2 mg atovaquone and 0.8 mg proguanil) was given for the treatment of P. chabaudi infection by oral gavage for 5 days. Pyrimethamine (100 μl, 0.2 mg/mouse) was given for the treatment of P. yoelii infection by intraperitoneal injection for 4 days. Naive recipient mice received 500 μl of the appropriate serum on days −1, 0, and 1 relative to the time of challenge on day 0. Immunized donor and naive mice (*n* = 5/group) were additionally challenged and acted as control groups.

### Immune depletion of CD4^+^ and CD8^+^ T cells.

BALB/c mice were depleted of CD4^+^, CD8^+^, or a combination of CD4^+^ and CD8^+^ T cells. Depleting antibodies (anti-CD4^+^ [GK1.5] and anti-CD8^+^ [53.5.8] antibodies; Bio X Cell, Lebanon, NH) were administered at 250 μg/mouse by intraperitoneal injection on days −2, −1, 4, and 8 relative to the time of challenge on day 0. Control mice received a dose of rat Ig control (Sigma-Aldrich) in parallel. Depletions were verified on days 1, 9, and 16 by staining spleen cells derived from unchallenged vaccinated mice with CD3 (clone 17A2 labeled with V450), CD4 (clone RM4-5 labeled with V500), and CD8 (clone 53.6.7 labeled with PerCp-Cy5.5) antibodies (BD Biosciences).

### Clinical study immunization protocol and treatment.

The clinical study was conducted in the Clinical Trial Unit, Griffith University, Gold Coast Campus, Southport, QLD, Australia. Study participants were healthy, RhD-positive, Caucasian males aged 18 to 50 years. Individuals were excluded if they had a history of malaria infection or had traveled to or lived (for >2 weeks) in a country where malaria is endemic during the previous 12 months. Other key eligibility criteria are listed on the Australian New Zealand Clinical Trials Registry (www.anzctr.org.au; study reference, ACTRN12615001126505).

The study utilized the P. falciparum 7G8 cell bank manufactured and characterized as previously described (54, 102). All processes were carried out in compliance with Annex 13 of the PIC/S Guide (104) in a clean room and monitored environment suitable for production of sterile biologics in accordance with approved protocols. A vial of the cell bank was retrieved from liquid nitrogen and taken to the clean room for processing. Thawing of the pRBCs, culture, and expansion of the parasite were undertaken as previously described with the following modification (54): leukocyte-depleted human blood group O RhD-positive erythrocytes (Key Biologics, LLC, Memphis, TN) were used during the manufacturing process.

On the day of inoculation, pRBCs from continuous cultures were harvested. Trophozoite/schizont-stage pRBCs were purified by magnetic separation over CS columns (Miltenyi Biotec, Bergisch Gladbach, Germany) on a VarioMACs magnet (Miltenyi Biotec) to limit the number of uninfected RBCs administered in an inoculum. The purity of the inoculum was >95% pRBCs. Cells were counted and resuspended in saline for injection to give a final volume of 2 ml/dose for intravenous administration, with each participant receiving 3 × 10^6^ pRBCs. The number of parasites present in the inoculum was verified retrospectively by qPCR assay of surplus material.

At approximately 1 h postinoculation, volunteers received treatment with doxycycline hydrochloride (100 mg, one capsule daily for 21 days). The parasite density was monitored on days 1 to 9 and every second day thereafter until day 37 and on day 90.

If the numbers of parasites in the blood increased exponentially and reached 11,550 parasites/ml (by qPCR) or clinical symptoms of malaria developed, rescue treatment with artemether-lumefantrine (A/L) was initiated and administration was according to its approved dosing schedule. If the participants had not received rescue treatment and were not qPCR positive by day 37, A/L treatment was initiated as a safety precaution.

### Quantification of P. falciparum in blood by qPCR.

Preparation of the sample, DNA extraction, and measurement of parasitemia by qPCR were conducted as previously described (102). A vial of lyophilized whole blood infected with a WHO P. falciparum international standard (NIBSC code 04/176) (103) was reconstituted in nuclease-free water. The reconstituted material was subsequently serially diluted and stored at −80°C for later use. The parasite concentration in participant blood (number of parasites per milliliter) was calculated based upon this standard, with 1 international unit (IU)/ml being equivalent to 0.5 parasites/ml (102). DNA was extracted using a QIAamp DNA blood minikit (Qiagen), with the manufacturer volumes being adjusted to extract 2 times the volume of blood. An internal control (equine herpes virus [EHV]) was added to the samples prior to DNA extraction to monitor the efficiency and reproducibility of the extraction process. Samples that amplified with threshold cycle values of <34 were considered efficiently extracted. For the qPCR primers and TaqMan MGB probe designs used, see the supplemental methods. The PCR mixtures contained a final concentration of 1× QuantiTect probe PCR master mix, 0.4 μM forward and reverse primers, and 0.16 μM TaqMan probe. The final reaction volume was 25 μl, and qPCR was performed under the following conditions: incubation at 95°C for 15 min, followed by 45 cycles of denaturation at 95°C for 15 s and annealing/extension at 60°C for 60 s.

### Isolation of peripheral blood mononuclear cells from clinical samples.

Whole blood was collected from the volunteers on days −1, 8, 15, 27, 37, and 90 (in relation to the time of inoculation on day 0) into sodium heparin tubes and centrifuged at 433 × *g* for 10 min. Plasma was removed and stored at −80°C for antibody analysis. To isolate PBMCs, density centrifugation with Ficoll-Paque (Amersham, Little Chalfont, UK) was used according to published methods (26). Cells were subsequently counted, with viability being determined using 0.4% trypan blue, and the cells were resuspended in freezing medium containing 90% heat-inactivated FBS and 10% dimethyl sulfoxide and frozen to −80°C at 1°C/min in freezing containers for 24 h (Nalgene, Rochester, NY), before transfer to liquid nitrogen for storage.

### Assessment of parasite-specific lymphocyte proliferative responses by flow cytometry.

Cells were thawed and washed in complete medium (RPMI 1640 containing 10% heat-inactivated human serum, 2 nM l-glutamine, 100 U/ml penicillin, 100 mg/ml streptomycin sulfate). Cells were resuspended in complete medium at a concentration of 2 × 10^6^ cells/ml. Prior to PBMC stimulation, the cell suspensions were stained with violet proliferation dye (VPD; labeled with V450; BD Biosciences) according to the manufacturer’s instructions. Cells were plated out at a concentration of 2 × 10^5^ cells/well in U-bottom 96-well plates. Control wells received 1% phytohemagglutinin (PHA; Gibco, Grand Island, NY) or medium. Experimental wells received freshly magnet-purified P. falciparum 7G8-infected trophozoite-stage RBCs (pRBCs) or uninfected RBCs at 6 × 10^5^ cells/well. The plates were incubated at 37°C in 5% CO_2_ for 7 days (26). For cytokine analysis, the culture supernatants were collected, stored at −80°C, and replaced with fresh medium on day 6.

After 7 days of culture, cells were pelleted at 1,200 rpm for 4 min, and sample replicates were pooled. Live/Dead Aqua dye (Thermo Fisher, Carlsbad, CA) was used according to the manufacturer’s instructions to assess cell viability. Cells were washed with fluorescence-activated cell sorting buffer before staining with γδ T cell receptor (TCR) (clone B11 labeled with PE-CF594; BD Biosciences) for 20 min on ice. Subsequently, the cells were washed and stained with CD3 (clone SK7 labeled with allophycocyanin-Cy7), CD4 (clone RPA-T4 labeled with FITC), and CD8 (clone RPA-T8 labeled with PE) for 20 min on ice (all from BD Biosciences). Cells were washed twice and resuspended for analysis on the LSR Fortessa flow cytometer and with FlowJo software (v10) (see Fig. S6 in the supplemental material).

### Statistics.

All data were statistically analyzed and graphed using GraphPad Prism software (v7) for Mac. Data are presented as the mean ± standard error of the mean (SEM), unless stated otherwise. An unpaired *t* test was used to compare peak parasitemia during challenge of the immunized and control groups. In some instances (e.g., passive serum transfer and T cell depletion), an area under the curve (AUC) analysis was conducted to compare the parasitemia curves between several groups. One-way or two-way analysis of variance (ANOVA) was performed on the data sets (e.g., the T cell proliferation data set), wherein 3 or more groups were compared, followed by Tukey’s or Dunnett’s multiple-comparison test. The Gehan-Breslow-Wilcoxon test was used to compare the differences in the survival curves. Correlation analysis was also conducted using GraphPad Prism software (v7) to generate Pearson’s coefficient (*r*) and *P* values. *P* values were considered significant when *P* was <0.05, <0.01, <0.001, or <0.0001, as indicated in the figures.

### Approval of animal and clinical studies.

All animal experiments were approved by the Griffith University Animals Ethics Committee (approval numbers GLY/05/12, GLY/04/16, and GLY/01/17).

The clinical study was approved by the Gold Coast Hospital and Health Services District Human Research Ethics Committee (HREC) and the Griffith University HREC. Griffith University was the study sponsor. The study was conducted in accordance with the principles of the Declaration of Helsinki and the standards of good clinical practice defined by the International Conference on Harmonization. An independent safety review team was appointed. Written informed consent was obtained from all the participants prior to the commencement of the study.

## Supplementary Material

Supplemental file 1
