# Supplementary material for: Controlled Infection Immunization Using Delayed Death Drug Treatment Elicits Protective Immune Responses to Blood-Stage Malaria Parasites
Source: Infect Immun. 2018 Dec 19;87(1):e00587-18. doi: 10.1128/IAI.00587-18 (PMC6300636; doi:10.1128/IAI.00587-18)
Supplement: Supplemental file 1 [file 3bc07453268b92df4b2ae7635d623cdf_IAI.00587-18-s0001.pdf]

Supplemental Figure Legends

Supplemental Fig 1. Persistence of *Plasmodium* spp. parasites during CIIs. BALB/c mice (n=10/group) underwent CII with 10^6 or 10^7 A) *P. chabaudi* or B) *P. yoelii*. Persistence was monitored during the 1st and 3rd CII over a 14-day period by qPCR. Data are expressed as mean ± SEM.

Supplemental Fig 2. Homologous challenge of mice receiving one CII with *P. chabaudi* or *P. yoelii*. BALB/c (n=10/group) and C57BL/6 (n=5-7/group) mice were administered 10^6 or 10^7 *P. chabaudi* (A, B) or *P. yoelii* (C, D) parasites and immediately started on doxycycline (50mg/kg) treatment over a 7-day period. Mice were challenged with 10^5 homologous parasites 4 weeks later. Parasitaemia was monitored every 2nd day post challenge. Data are expressed as mean ± SEM. † indicates number of mice that were euthanized. Differences in peak parasitaemia was assessed by unpaired t-test. *** $p<0.001$, ****$p<0.0001$, ns – not significant.

Supplemental Fig 3. Clinical scores of mice receiving 1 or 3 CIIs with *P. chabaudi* or *P. yoelii* after homologous challenge. BALB/c (A-D) and C57BL/6 (E-H) mice were administered 10^5 -10^7 parasites *P. chabaudi* or *P. yoelii* and immediately started on doxycycline (50mg/kg) treatment over a 7-day period. Mice were challenged with 10^5 homologous 4 weeks after the last day of treatment and health was monitored by assessing symptoms of disease based on clinical score criteria. Data are expressed as mean ± SEM. † indicates number of mice that were euthanized.

Supplemental Fig 4. Clinical scores and haemoglobin monitoring of CII mice receiving heterologous challenge. BALB/c (A-B) and C57BL/6 (C-D) mice were administered 10^7 parasites *P. chabaudi* (A, C) or *P. yoelii* (B, D) and immediately started on doxycycline (50mg/kg) treatment over a 7-day period. Mice were challenged with 10^5 heterologous parasites 4 weeks after receiving 3 CIIs and health was monitored by i) assessing symptoms of disease and scoring mice based on criteria listed on the clinical score sheet and ii) haemoglobin concentration. Data are expressed as mean ± SEM. † indicates number of mice that were euthanized.

Supplemental Fig 5. Example gating strategy for assessing activation of $CD4^+$ and $CD8^+$ T cells in peripheral blood collected from mice 7 days after an CII.

Supplemental Fig 6: Example gating strategy for assessment of lymphocyte proliferation using VPD.

Supplemental Fig 1.

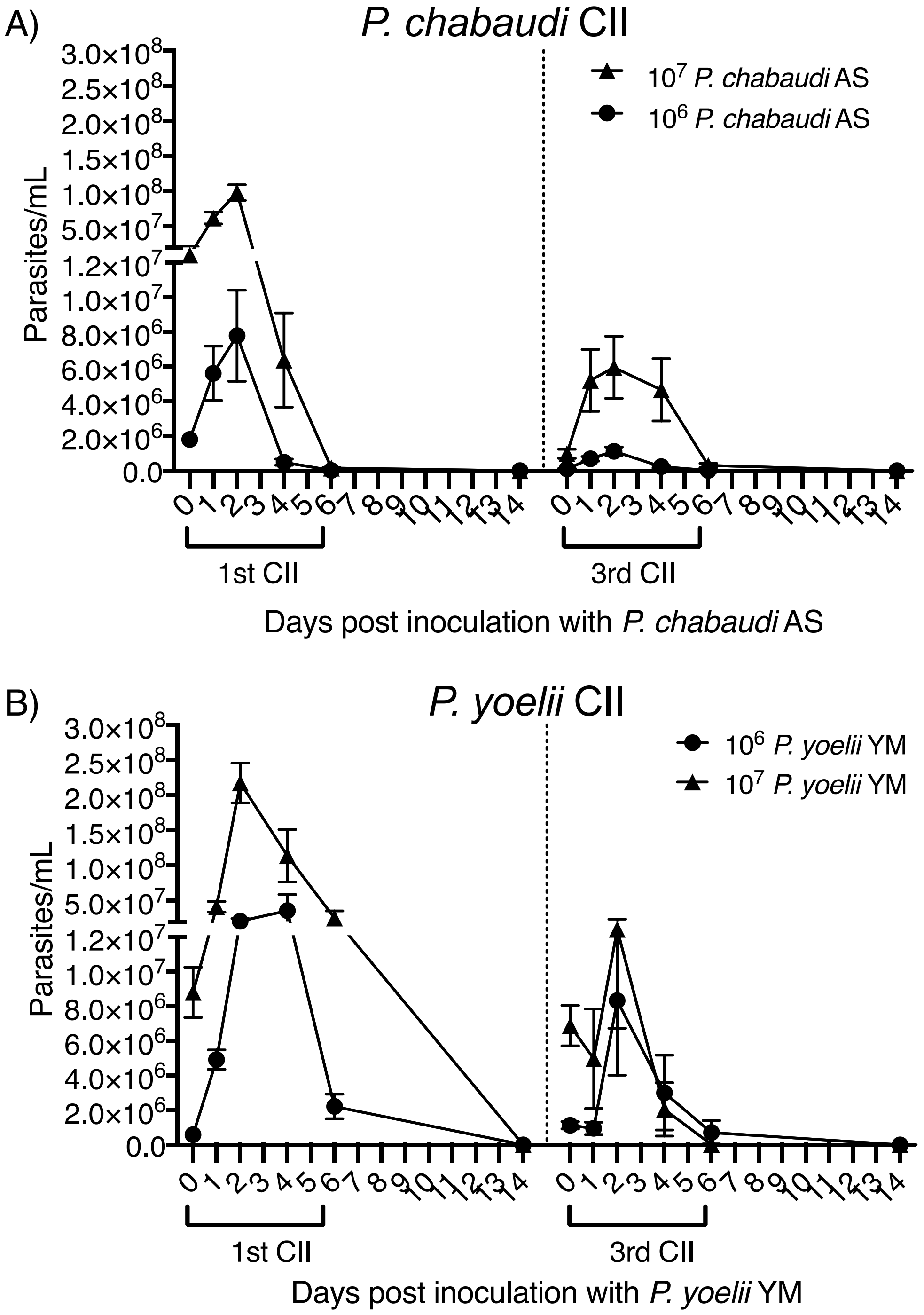

Supplemental Fig 2.

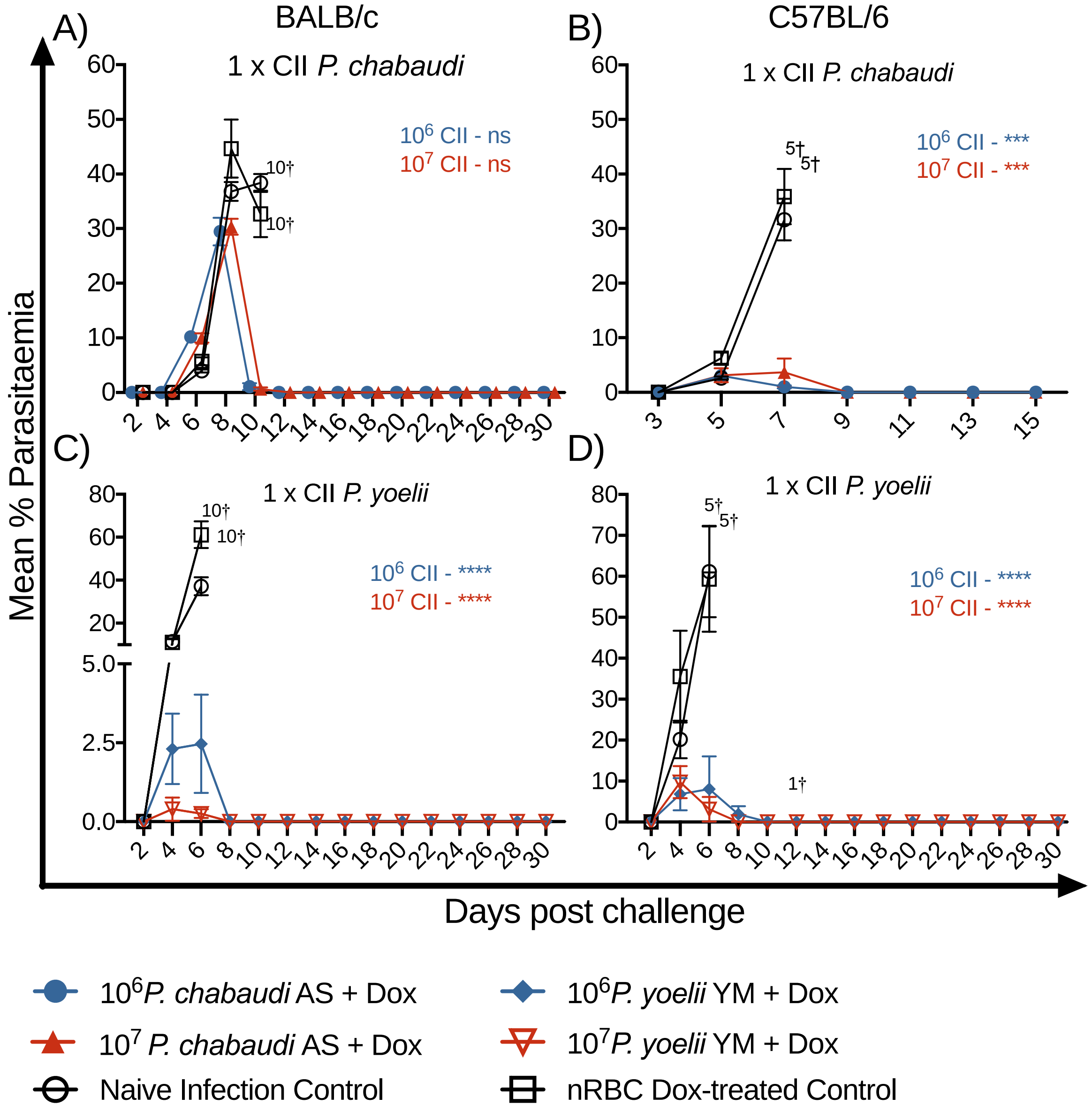

Supplemental Fig 3.

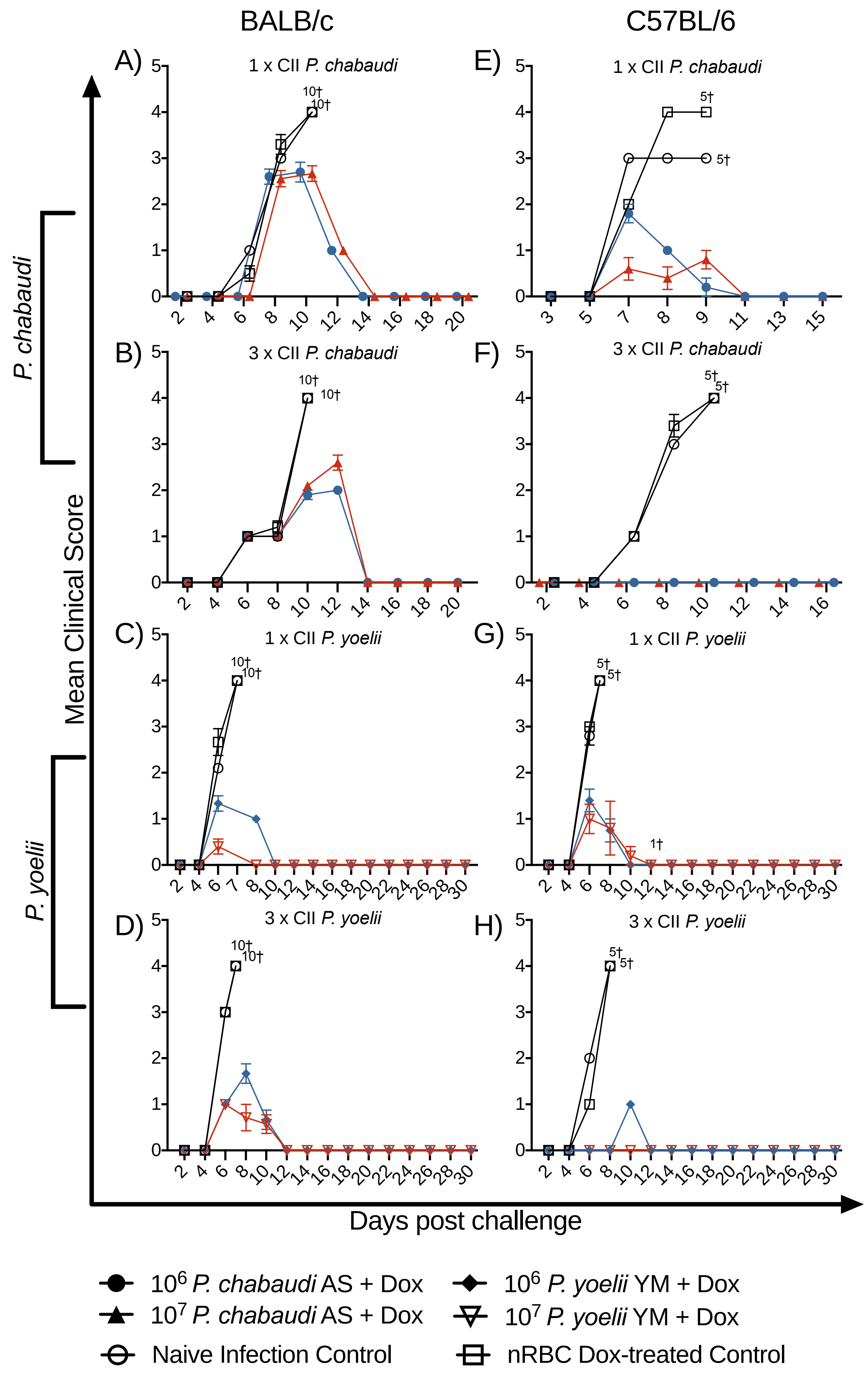

47 **Supplemental Fig 4.**

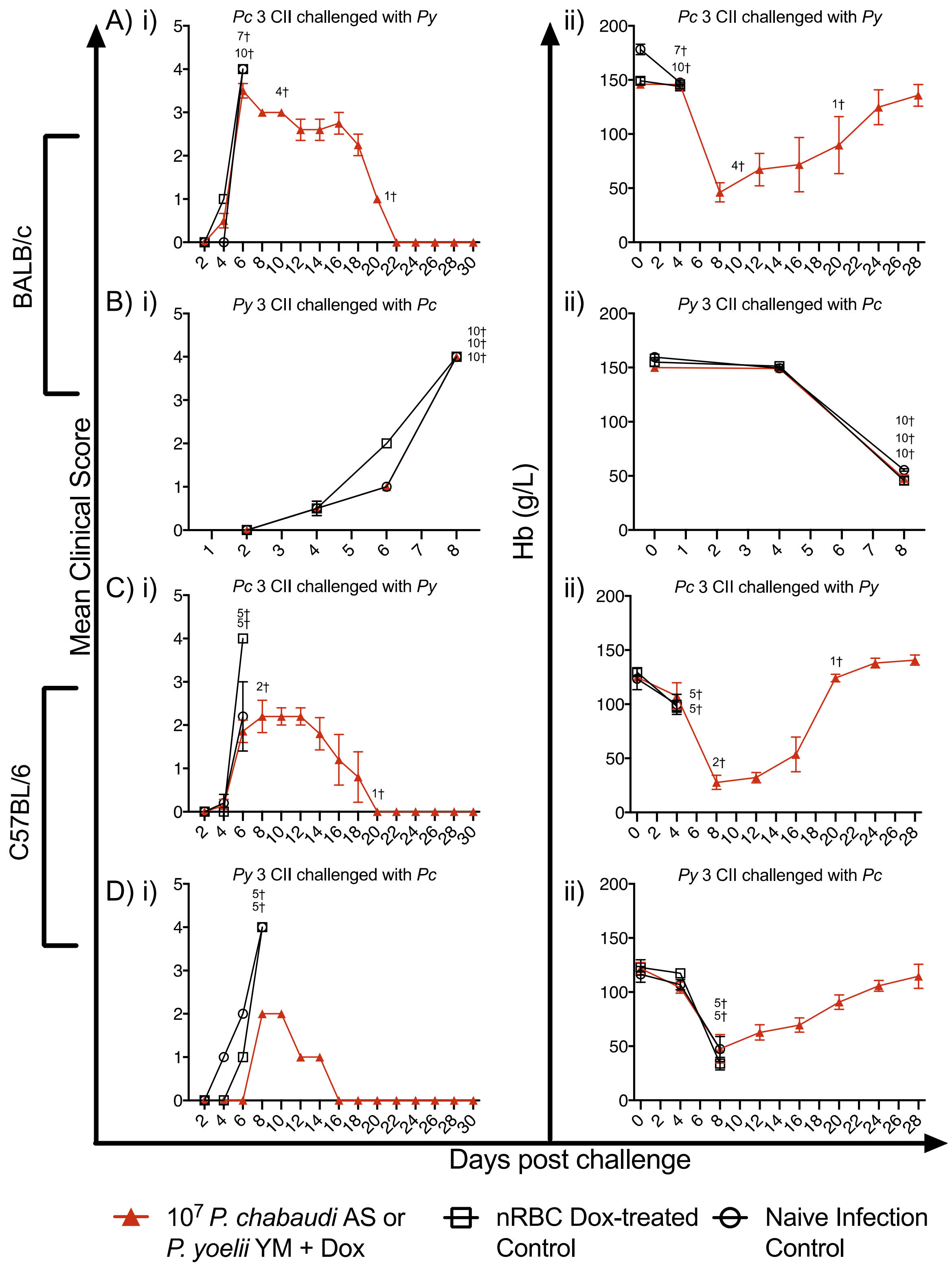

48

49 **Supplemental Fig 5.**

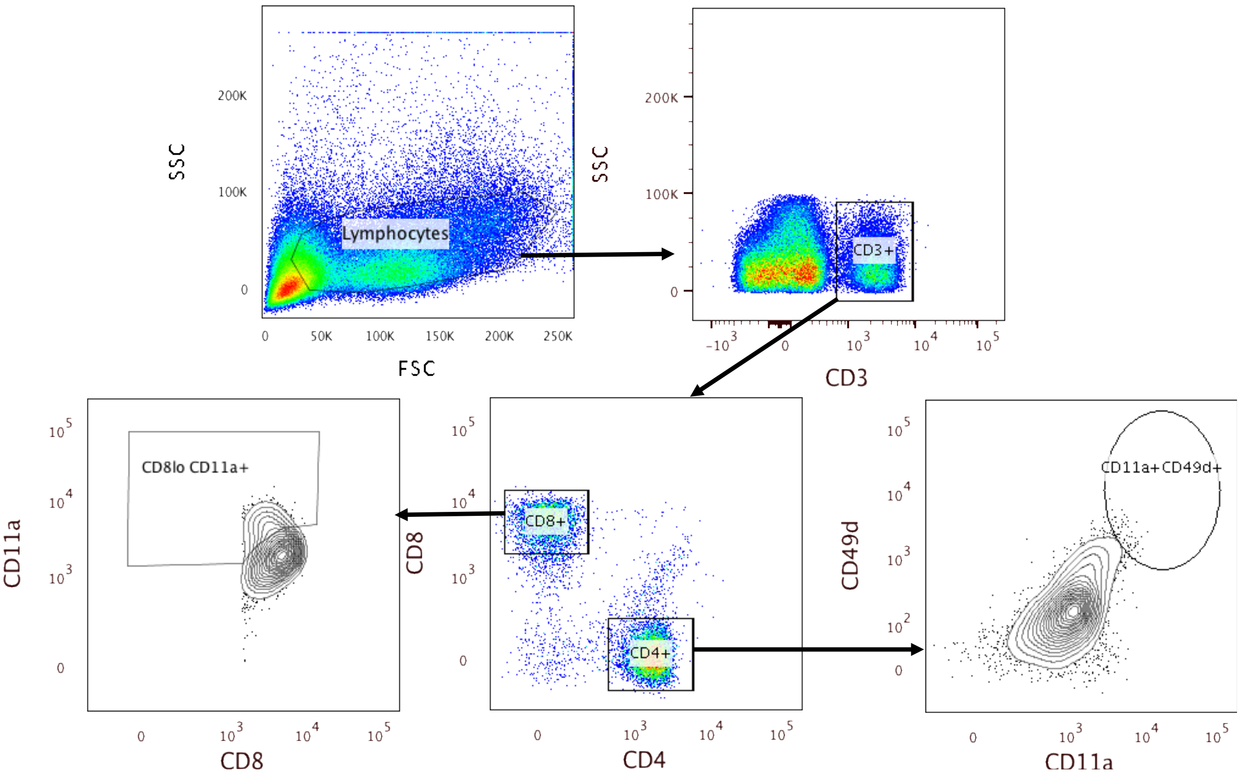

50

51

Supplemental Fig 6.

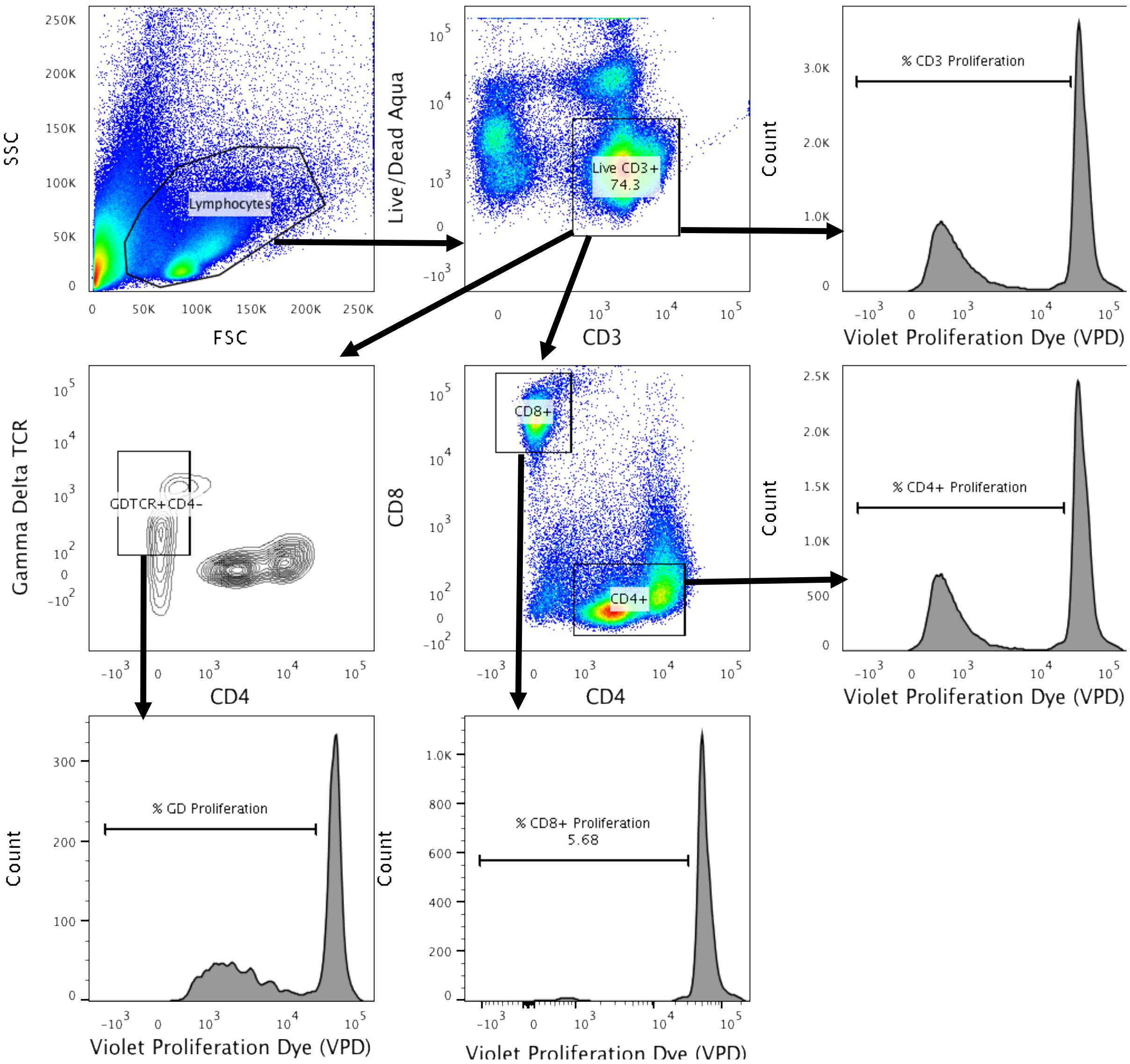

Supplemental Material 1: qPCR primers and probes for detection of *P. falciparum* 7G8

Primers (Sigma-Aldrich)

18SPFR-F 5’- CTTTTGAGAGGTTTTGTTACTTTGAGTAA-3'
18SPFR-R 5'-TATTCCATGCTGTAGTATTCAAACACA-3'

Internal control for efficiency of DNA extraction
EHV-330F5'-GATGACACTAGCGACTTCGA-3'
EHV-410R5'- CAGGGCAGAAACCATAGACA-3'

TaqMan Probes (Applied Biosystems/Biosearch Technologies)

EHV-360 FAM- 5'-TTTCGCGTGCCTCCTCCAG -3'-BHQ1
18SPFR-F FAM-5’- TGTTCATAACAGACGGGTAGTCATGATTGAGTTCA -3'- BHQ1

Supplemental Table 1. Production of Th1, Th2 and Th17 cytokines in response to homologous and heterologous live pRBCs in BALB/c CII mice.

Cytokine		Cytokine concentration (pg/ml ± SEM)							
		3 x CII with *P. chabaudi* AS				3 x CII with *P. yoelii* YM			
		Homologous		Heterologous		Homologous		Heterologous	
		Control	Immunized	Control	Immunized	Control	Immunized	Control	Immunized
Th1	IFNγ	1.20 ± 0.03	32.80 ± 8.03 *	1.24 ± 0.14	37.06 ± 19.52 **	0.42 ± 0.32	143.33 ± 41.96 ****	0.21 ± 0.21	0.37 ± 0.36
	IL-2	0.77 ± 0.44	47.13 ± 14.73 ***	0.97 ± 0.57	24.11 ± 8.78	0.85 ± 0.85	29.54 ± 1.54 ****	0.00 ± 0.00	1.08 ± 1.08
	TNF	0.00 ± 0.00	28.88 ± 5.82 ***	0.72 ± 0.72	26.70 ± 10.07 **	5.69 ± 2.35	46.06 ± 4.39 ****	2.73 ± 1.59	6.75 ± 3.59
Th2	IL-4	0.00 ± 0.00	0.00 ± 0.00	0.00 ± 0.00	0.00 ± 0.00	0.48 ± 0.24	5.31 ± 1.35 ****	0.54 ± 0.18	0.15 ± 0.07
	IL-6	1.76 ± 1.40	31.81 ± 14.08	6.26 ± 4.58	29.24 ± 15.36	9.51 ± 3.62	27.53 ± 4.60 ****	4.06 ± 1.02	4.70 ± 0.99
	IL-10	6.16 ± 1.35	36.95 ± 14.35 **	4.38 ±1.99	27.75 ± 8.93 *	1.16 ± 1.16	28.59 ± 4.65 ****	0.00 ± 0.00	0.00 ± 0.00
Th17	IL-17A	0.00 ± 0.00	0.00 ± 0.00	0.00 ± 0.00	0.00 ± 0.00	0.27 ± 0.14	26.31 ± 12.97 **	0.35 ± 0.21	0.44 ± 0.18

Culture supernatants from parasite stimulated splenocyte proliferation assays were collected after 54h and cytokines were quantified. The supernatant was pooled from triplicate wells for each spleen. Mice received 3 CIIs with 10^7 *P. chabaudi* or *P. yoelii* pRBCs (Immunized) or an equivalent dose of nRBCs under doxycycline treatment (Control). Each group contained n=3 mice. Data represents mean ± SEM and were analysed by two-way ANOVA followed by Tukey's multiple comparison test; * p<0.05, ** p<0.01, *** p<0.001, **** p<0.00001

.

Supplemental Table 2. Production of Th1, Th2 and Th17 cytokines in response to homologous and heterologous live pRBCs in C57BL/6 CII mice.

Cytokine		Cytokine Concentration (pg/ml ± SEM)							
		3 x CII with *P. chabaudi* AS				3 x CII with *P. yoelii* YM			
		Homologous		Heterologous		Homologous		Heterologous	
		Control	Immunized	Control	Immunized	Control	Immunized	Control	Immunized
Th1	IFNγ	7.48 ± 3.10	217.17 ± 32.80 ****	5.79 ± 3.31	209.71 ± 47.64 ****	0.18 ± 0.18	93.85 ± 27.16 ****	0.18 ± 0.11	22.40 ± 7.79
	IL-2	0.97 ± 0.46	6.66 ± 0.68 ****	0.67 ± 0.40	8.58 ± 1.60 ****	0.56 ± 0.56	5.44 ± 1.00 **	0.56 ± 0.56	1.12 ± 0.35
	TNF	54.69 ± 18.42	135.65 ± 28.02	49.20 ± 17.79	141.10 ± 29.51	0.94 ± 0.63	236.11 ± 102.25 **	6.36 ± 1.53	52.39 ± 13.69
Th2	IL-4	0.09 ± 0.09	0.27 ± 0.09	0.03 ± 0.03	0.33 ± 0.25	0.60 ± 0.27	0.32 ± 0.14	0.25 ± 0.21	0.81 ± 0.24
	IL-6	32.57 ± 11.01	96.55 ± 26.32 *	20.74 ± 4.99	56.48 ± 15.03	2.75 ± 0.58	36.81 ± 11.84 ***	3.18 ± 1.19	12.28 ± 4.48
	IL-10	2.95 ± 1.92	31.62 ± 7.24 ***	4.97 ± 2.93	32.17 ± 10.37 **	7.12 ± 3.58	5.07 ± 5.07	17.36 ± 10.01	1.25 ± 0.79
Th17	IL-17A	0.05 ± 0.05	0.35 ± 0.19 *	0.05 ± 0.03	0.04 ± 0.03	0.29 ± 0.11	1.74 ± 0.60 **	0.73 ± 0.18	0.19 ± 0.10

Culture supernatants from parasite stimulated splenocyte proliferation assays were collected after 54h and used in cytokine bead arrays to quantify the level of cytokines produced after stimulation with homologous or heterologous pRBCs. The supernatant was pooled from triplicate wells for each spleen. Mice received 3 CIIs with 10^7 *P. chabaudi* or *P. yoelii* pRBCs (Immunized) or an equivalent dose of nRBCs under doxycycline treatment (Control). Each group contained n=3 mice. Data represents mean ± SEM and were analysed by two-way ANOVA followed by Tukey's multiple comparison test; * $p<0.05$, ** $p<0.01$, *** $p<0.001$, **** $p<0.00001$

Supplemental Table 3. Adverse events reported during clinical study.

Symptom	Severity-Mild	Severity-Moderate	Severity-Severe	Total
Headache	4	0	0	4
Nausea	2	0	0	2
Subjective fever	2	0	0	2
Buzzing in head/hot flush	1	0	0	1

*Only those adverse events deemed possibly related to administration of the inoculum are reported.
